# AKR1C3 enhances radioresistance in esophageal adenocarcinoma via inhibiting ferroptosis through suppressing TRIM21-mediated ubiquitination of HSPA5

**DOI:** 10.1038/s41419-025-07773-z

**Published:** 2025-07-02

**Authors:** Feng Ju, Jialei Weng, Ningbo Fan, Zhefang Wang, Chenghui Zhou, Xinlei Zhao, Nellie Horstmann, Xiaolin Wu, Sascha Hoppe, Bo You, Keying Li, Jianxin Duan, Margarete Odenthal, Axel M. Hillmer, Alexander Quaas, Christiane J. Bruns, Yue Zhao

**Affiliations:** 1 Department of General-, Visceral-, Thoracic- and Transplantation Surgery, University Hospital of Cologne, Cologne, Germany; 2 Department of Surgical Oncology, Sir Run Run Shaw Hospital, School of Medicine, Zhejiang University, Hangzhou, PR China; 3 Department of Plastic and Reconstructive Surgery, Second Affiliated Hospital, School of Medicine, Zhejiang University, Hangzhou, PR China; 4 Institute of Pathology, Faculty of Medicine and University Hospital of Cologne, Cologne, Germany; 5 Institute of Otolaryngology Head and Neck surgery, Affiliated Hospital of Nantong University, Nantong, PR China; 6 Ascentawits Pharmaceuticals Ltd, Shenzhen, PR China; 7 Center for Molecular Medicine Cologne, University of Cologne, Cologne, Germany

**Keywords:** Oesophageal cancer, Radiotherapy, Oesophageal cancer

## Abstract

Esophageal adenocarcinoma (EAC) is the predominant subtype of esophageal cancer (EC) in high-income countries, and radioresistance is one of the key factors for the poor prognosis. In this study, we successfully established a radioresistant EAC in vitro model. Aldo-keto reductase 1C3 (AKR1C3) was identified as a promising regulator of radioresistance by RNA-seq analysis and subsequent functional studies. Through integrated analyses of scRNA-seq and TCGA datasets, we found that AKR1C3 was likely to enhance radioresistance by inhibition of ferroptosis. Indeed, analysis of the lipid ROS level by C11-Bodipy staining and the result of transmission electron microscopy revealed that AKR1C3 could prevent EAC cells from ferroptosis. Mechanistically, AKR1C3 binds to the nucleotide-binding domain of HSPA5, thereby inhibiting the E3 ligase TRIM21-induced ubiquitin-dependent proteasomal degradation of HSPA5, which further stabilizes GPX4, thus inhibiting ferroptosis. Importantly, AKR1C3 inhibitor resensitized the EAC patient-derived organoids to radiotherapy. In conclusion, this study highlights AKR1C3 as a regulator of radioresistance and a potential therapeutic target in EAC.

## Introduction

Esophageal cancer (EC) ranks as the 7th highest cause of mortality (estimated 445,000 deaths) and 11th highest in incidence (estimated 511,000 new cases) worldwide in 2022 [1]. Esophageal adenocarcinoma (EAC) is one of the major histological subtypes of esophageal cancer [2]. The ChemoRadiotherapy for Oesophageal Cancer followed by Surgery Study (CROSS), which compared neoadjuvant chemoradiotherapy plus surgery to surgery alone in patients with EAC, demonstrated a notable survival advantage in the former group [3]. Recently, a real-world study from the Netherlands further underscored the potential of the CROSS regimen for patients with resectable esophageal or gastro-esophageal junction adenocarcinoma in clinical routine [4]. Despite significant advancements in diagnosis and treatment over recent decades, the global 5-year survival rate for patients with EAC remains below 15% [5]. The major causes of the low survival are tumor recurrence due to therapy resistance [6]. Recent advancements in molecular mechanism research, including non-coding RNAs, immunomodulation, and cancer stemness, have shed light on the underlying factors contributing to radioresistance in EAC [7–10]. However, addressing radiotherapy resistance still requires further in-depth research, particularly in identifying effective treatment targets.

Aldo-keto reductases (AKRs), a superfamily of NADP(H)-dependent oxidoreductases, are primarily located in the cytoplasm across various phyla, typically existing as monomers with a molecular weight ranging from 34 to 37 kDa [11]. In living organisms, the primary role of AKRs is to facilitate the reduction of carbonyl compounds to their corresponding alcohols. Specifically, AKRs catalyze the conversion of aldehydes into primary alcohols and ketones into secondary alcohols [12]. AKRs families and subfamilies are delineated through sequence alignment, organizing related members based on protein function [13]. AKR1C3 is one of the most extensively studied members of the AKRs. Its primary role involves serving as a prostaglandin (PG) F_2_α synthase, catalyzing the reduction of PGD_2_ and PGH_2_ to PGF_2_α [14]. AKR1C3 participates in NF-κB signaling and IL6/STAT3 pathway, resulting in cell proliferation and metastasis in hepatocellular carcinoma (HCC) cells, while treatment with the AKR1C3 inhibitors could suppress tumor growth and promote cell death [15]. AKR1C3 has also been observed to regulate lipid droplet formation in HCC, consequently contributing to sorafenib resistance, while its inhibition rapidly induces mitochondrial fission and apoptosis, implying AKR1C3 as a promising therapeutic target in HCC [16]. Another study has investigated AKR1C3 inhibitors, with co-administration notably re-sensitizing doxorubicin (DOX) in the resistant breast cancer cell lines [17]. In castration-resistant prostate cancer, AKR1C3 has been validated as a potential upstream regulator in JNJ-pan-AR resistant cells, with overexpression of AKR1C3 contributing to anti-androgen therapy resistance [18]. AKR1C3 also plays a crucial role in redox homeostasis by limiting the accumulation of ROS through the modulation of NADP(H)-dependent pathways, thereby maintaining cellular redox balance [19, 20]. Consequently, AKR1C3-driven redox regulation increases therapeutic resistance by protecting cancer cells from ROS-induced cell death [16, 21]. Further investigation into the molecular function of AKR1C3 in redox modulation, cell death, and its impact on radioresistance may hold potential value for improving the prognosis of EAC patients.

Recent studies have provided compelling evidence supporting the role of redox regulation in cancer progression and therapy resistance, particularly through ferroptosis [22, 23]. Ferroptosis is an iron-dependent form of regulated cell death and is primarily triggered by phospholipid peroxidation, characterized by notable morphological alterations such as decreased mitochondrial cristae, rupture of the bilayer phospholipid membrane, and condensation of the mitochondrial membrane [24]. The balance of lipid antioxidant metabolism is maintained by the intricate interplay of various factors, including glutathione peroxidase 4 (GPX4), coenzyme Q10 (CoQ), ferroptosis inhibitor protein 1 (FSP1), and dihydroorotate dehydrogenase (DHODH) [25–28]. GSH serves as a crucial reductant in organisms, capable of converting phospholipid hydroperoxides (L-OOH) to phospholipid alcohols (L-OH) with the catalytic assistance of GPX4, thereby preventing the cells from ferroptosis [29]. In recent years, the role of ferroptosis in radiotherapy resistance has been extensively explored in various oncology studies [30–34]; however, its implications in EAC remain unknown. Moreover, a recent study suggests that AKR1C3 could inhibit ferroptosis via YAP/SLC7A11 axis in HCC [35]. Whether AKR1C3 regulates ferroptosis in EAC requires further investigation.

In this study, we aimed to investigate the radioresistance of EAC and the underlying mechanisms. We established the radioresistant EAC cell line and found that AKR1C3 was significantly upregulated in the radioresistant cells by RNA-seq. Knockdown/inhibition of AKR1C3 resensitized EAC cells/patient-derived organoids to radiotherapy. Mechanistically, we revealed a novel role of AKR1C3 in mediating ferroptosis. AKR1C3 could bind to the nucleotide-binding domain of Heat-shock-protein family A member 5 (HSPA5) and reduce the interaction between HSPA5 and tripartite motif-containing protein 21 (TRIM21) to inhibit the ubiquitin-dependent proteasomal degradation of HSPA5, which could further stabilize GPX4 and inhibit ferroptosis. Based on the above, our findings suggest that AKR1C3 inhibits ferroptosis by stabilizing the HSPA5/GPX4 axis, thereby enhancing radioresistance in EAC.

## Materials and methods

### Cell lines and clinical samples

The human EAC cell line SKGT-4 was obtained from Deutsche Sammlung von Mikroorganismen und Zellkulturen (DSMZ, Germany), OE33 was obtained from the Sigma Cell Line Bank (Sigma, #96070808, Germany). All the EAC cells were maintained in RPMI1640 medium (Life Technology, Germany) with 10% fetal bovine serum (FBS) (Capricorn, Germany), penicillin and streptomycin (100 U/mL penicillin + 0.1 mg/mL streptomycin) (PAN Biotech, Aidenbach, Germany) in a humidified atmosphere of 5% CO_2_ at 37 °C. HEK293T cells (Sigma, #12022001) were maintained in DMEM high-glucose medium with 10% FBS, 2 mM L-Glutamine (Invitrogen, USA), penicillin, and streptomycin (100 U/mL penicillin + 0.1 mg/mL streptomycin) in a humidified atmosphere of 5% CO_2_ at 37 °C. Furthermore, all cell lines were tested and confirmed free from mycoplasma contamination.

A total of eight paired samples were collected from EAC patients at University Hospital of Cologne for immunohistochemistry. Four naïve patients who were pathologically diagnosed with EAC at University Hospital of Cologne were investigated for scRNA-seq in this study. Written informed consent was obtained from all patients, and the study was approved by the Institutional Ethics Committee of the University Hospital of Cologne (ID: 13-091 and ID:18-274).

### Establishment of the radioresistant EAC cell line

OE33 was selected for the establishment of the radioresistant cell line. Two flasks of OE33 cells from the same passage were labeled as OE33P (the parental cell line) and OE33R (the radioresistant cell line). OE33R received 2 Gy gamma ray irradiation (BIOBEAM GM 8000, Gamma-Service Medical GmbH, Germany) once it reached 50–70% confluence. OE33P was mock-irradiated (in order to ensure the same environment as OE33R). Cells were passaged when they reached 90% confluence. Generally, a rest period of 7–10 days was required between consecutive irradiation treatments. Upon completion of 25 cycles, amounting to a total dose of 50 Gy, the cells underwent a 1-week rest period. Subsequently, we performed a single-cell colony formation experiment on OE33R, and after 1 week, a healthy colony was selected as the radioresistant model of OE33R. Throughout the establishment process, OE33P was maintained under identical environmental and experimental conditions as OE33R to ensure the accuracy and reliability of the model.

Colony formation was performed to validate the radioresistant model, the data were fitted into the single-hit multi-target formula: S = 1 − (1 − e^−D/D0^)^N^. S is the fraction of cells surviving, D_0_ is the “mean lethal dose”, which required to reduce the survival of a cell population to 37% of its initial value. Dq is the quasi-threshold dose, the width of the “shoulder,” and correlates with repair capacity. N is the extrapolation number [36].

### Colony formation assay

EAC cells (300–2000 cells per well) were seeded in 6-well plates with 2 mL full RPMI 1640 medium each well at 37 *°*C with 5% CO_2_. The next day, experimental groups underwent irradiation or treatment with related agents, while control groups were under mock treatment. After 7–14 days, when the complete colonies were formed, the growth medium was removed. The colonies were then washed once with PBS and fixed with formalin at room temperature for 30 min. Subsequently, the colonies were stained with crystal violet (Sigma-Aldrich, Germany) for 20 min. After staining, the colonies were gently washed twice with water and allowed to air dry. Colonies containing more than 50 cells were identified and recorded under the microscope (Leica, DMIL, Germany).

### RNA-seq and data analysis

Triplicates of OE33P and OE33R cell lines were cultured and maintained under optimal conditions. RNA extraction was performed using the AllPrep DNA/RNA/Protein Mini Kit. Subsequently, the extracted RNA samples were sent to Macrogen Europe (Amsterdam, Netherlands) for total RNA sequencing by the Illumina NovaSeq 6000 platform. Raw data quality was assessed using FastQC. Trimmomatic was used for trimming reads and removing adapter sequences. BWA-MEM, Samtools, and FeatureCounts were applied for downstream analyses. The following R packages were used for further data analyses and the graphing of volcano plot, heatmap, and dotplot: ggplot2, ggrepel, ggpubr, DOSE, clusterProfiler, org.Hs.eg.db, enrichplot, pathview, ggnewscale, pheatmap, DESeq,2 and dplyr. The RNA-seq results were included in the Supplementary RNA-seq.

### Quantitative real-time PCR (qRT-PCR)

TRI reagent (Sigma-Aldrich, Germany) was used for extracting total RNA from the cultured EAC cells. Subsequently, the High-Capacity cDNA Reverse Transcription Kit (Applied Biosystems, Thermo Fisher Scientific) was used for cDNA synthesis according to the manufacturer’s protocol. Relative expression of target mRNAs was measured by using Fast SYBR Green Master Mix (Invitrogen) with QuantStudio 7 Flex (Applied Biosystems, Thermo Fisher Scientific) and analyzed by the delta-delta-CT method.

Primer sequences (5’–3’):

GAPDH-for GAAGGTGAAGGTCGGAGTC

GAPDH-rev GAAGATGGTGATGGGATTTC

hAKR1C3-for GTCATCCGTATTTCAACCGGAG

hAKR1C3-rev CCACCCATCGTTTGTCTCGTT

### Western blot

Cells were harvested and lysed with cell lysis buffer (Cell Signaling #9803) supplemented with 1 mM phenylmethylsulfonyl fluoride (PMSF). Later, cell lysates were sonicated for 5 min, then centrifuged at 13,000 rpm for 10 min at 4 °C. The supernatant was collected, and the protein concentration was then measured by BCA protein assay (Thermo Fisher Scientific). Protein was cooked with bolt loading buffer in 1× NuPAGE LDS sample buffer (Invitrogen) at 70 °C for 10 min. Twenty microgram protein samples were electrophoresed for 70–120 min in the SDS-PAGE gel (Tris-Glycine, self-made) and transferred to PVDF membrane (MA-CHEREY-NAGEL, Germany) by semi-dry electroblotting (Bio-Rad, Singapore). The membranes were blocked for 1 h in Roti-Block buffer (Carl Roth, Germany) at room temperature on the shaker, then incubated with specific primary antibodies at 4 °C overnight on the rotator. Membrane was incubated with HRP-conjugated secondary antibody (Invitrogen #31430 and 31460; Cell Signaling #93702 Light-Chain Specific) for 1 h at room temperature and visualized with SuperSignal West Pico PLUS Chemiluminescent Substrate (Thermo Fisher Scientific, USA) and detected by ChemoStar ECL Imager (Intas Science Imaging, Germany). The original western blot images are available in the Supplementary Material.

### Plasmid constructs and transfection

The pLVX puro TRIM21-GFP was a gift from Gaudenz Danuser (Addgene plasmid # 116941; http://n2t.net/addgene:116941; RRID:Addgene_116941). The concentration of pLVX puro TRIM21-GFP was 1 μg/mL, and incubation for 72 h, an empty pLVX puro vector was used for the control group. AKR1C3 plasmid constructs and transfection details were mentioned in our previous study [20]. The Flag-tagged AKR1C3 plasmid, HA-tagged full-length and truncated HSPA5 plasmids were designed and synthesized by RiboBio (Guangzhou, China). The concentration of the truncated plasmids was 1 μg/mL, and incubation for 48 h. Lipofectamine™ 3000 (L3000001, Invitrogen) was used for the transfection.

### RNA interference

siRNAs were purchased from siTOOLs Biotech GmbH. EAC cells were seeded in 6-well plates overnight. After 24 h, siRNAs were added into the wells at a final concentration of 1–20 nM, negative control siRNA was used for the control group. Lipofectamine™ 3000 was used for the transfection. With 72 h co-culture, cells were harvested for the subsequent experiments.

### Immunofluorescence

EAC cells were seeded in 8-well chamber slides (IBIDI #80806) with 20–40k cells/well and incubated at 37 °C with 5% CO_2_ for 24 h. Then the test groups were irradiated with 3 or 8 Gy. After an additional 24 h, the cells were gently washed twice with PBS. The cells were then fixed with 4% formalin for 15 min, followed by three washes with PBS for 5 min each. Permeabilization was achieved by treating the cells with 0.2% Triton X-100 for 15 min, followed by three washes with PBB (PBS + 0.5% BSA) for 5 min each. The cells were then blocked with normal serum block for 40 min at room temperature and washed once with PBB for 5 min. Then cells were incubated with primary antibody of anti-H2AX (1:800) overnight at 4 °C. The following morning, cells were washed with PBB 5 min for 3 times and incubated with secondary antibody (Alexa 488-conjugated anti-mouse, 1:1000) for 1 h at room temperature. DAPI (1:4000) was used for nuclear staining. Afterwards, wash cells 5 min for 3 times, cover slides with mountain medium and keep them in darkness. Images were taken with an IX83 inverted microscope.

For the co-localization experiments, the primary antibodies were anti-HSPA5 (1:50, anti-Mouse, Proteintech #666574) and anti-TRIM21 (1:50, anti-Rabbit, Cell Signaling #92403), the secondary antibodies were anti-Mouse Alexa Fluor™ Plus 488 (1:200, Invitrogen #A32723) and anti-Rabbit Alexa Fluor™ 594 (1:200, Invitrogen #A11012). Images were taken with STELLARIS 5 Confocal Microscope (Leica) and processed by ImageJ.

### Comet assay

Slides were first coated with Agarose Normal Melt (Merck, #9012-36-6). A mixture of 1500 cells in 5 μL of medium was prepared and combined with 50 μL of Agarose Normal Melt (#6351.1, Carl Roth) at 37 °C. This mixture was then carefully dropped onto the prepared slide. The slide was subsequently incubated at 4 °C for 20 min before being placed into lysis buffer overnight at 4 °C. The following day, the slide was positioned in the electrophoresis tank and run for 20 min at 30 V. After electrophoresis, the slide was immersed in 70% alcohol for 30 min, followed by drying. Subsequently, Nancy-520 staining was performed for 30 min. Images were taken with IX83 inverted microscope.

### Immunohistochemistry (IHC)

Pre-neoadjuvant biopsies are endoscopic specimens that have not received neoadjuvant treatment, and postoperative tumor tissues are postoperative specimens that have undergone CROSS treatment. The criteria of the response evaluation was described in our previous study [34], the major response group included grade 3 and 4 (less than 10% vital residual tumor cells or complete response), and the minor group included grade 1 and 2 (more than 10% vital residual tumor cells). Briefly, slides were incubated at 60 °C 1 h, then slides were deparaffinized with xylene/2-propanol/ethanol, then 97 °C 20 min for antigen retrieval in citrate buffer (pH 6.0). Slides were put into the 0.3% H_2_O_2_ in methanol for 20 min to inactivate endogenous peroxidases. The antibodies used are listed in Supplementary Table S1. H-score referred to the “quickscore” method [37].

### scRNA-seq and data analysis

Tissue was washed with PBS and dissociated with ophthalmic scissors to finely cut pieces of 1–2 mm. The pieces were digested in 2 mL sCellLive™ Tissue Dissociation Solution (Singleron Biotechnologies, #1190062) at 37 °C for 15 min with continuous agitation on a thermal shaker. Following digestion, the suspension was filtered using a 40-µm sterile strainer (Greiner, #42040). The cells were centrifuged at 350 × *g* for 5 min at 4 °C and the cell pellets were resuspended in 1 mL PBS. The cells were counted using acridine orange/propidium iodide with a Luna FX7 automated cell counter (Logos Biosystems, Villeneuve d’Ascq, France).

The single-cell RNA-seq libraries were constructed using GEXSCOPE™ Single Cell RNAseq Library Kit (Singleron Biotechnologies, #4180011) and then were sequenced on an Illumina NovaSeq 6000 using a 2 × 150-bp approach to a final depth of 90 GB per library. The reads were demultiplexed according to the multiplexing index sequencing on Illumina’s BaseCloud platform. Fastq files were preprocessed using the CeleScope® tools (1.10.0; www.github.com/singleron-RD/CeleScope; Singleron Biotechnologies GmbH), using the default parameters except that the poly-A filter was switched off. The gene count matrix was then generated, providing the number of unique molecular identifiers (UMI) for each gene and cell.

Gene count matrices were analyzed using R (v. 4.2.2) and the Seurat library (v. 4.3.0). The cells were filtered according to the number of features (more than 200 but less than 5000), the proportion of reads from mitochondria (less than 50%) and the number of UMI counts (more than 500 UMIs). The features, which were not present in at least three cells, were filtered out. The datasets were then integrated, normalized after which the highly variable genes were identified and scaled. Principal component analysis (PCA) was performed and 30 npcs were used for the clustering process with the FindClusters function and for uniform manifold approximation and projection (UMAP) embedding. Cell type annotation: Cancer cells: “EPCAM”; Proliferating cancer cells: “MKI67”, “EPCAM”; Late erythroid: “HBA1”; Immune cells: “PTPRC”; Macrophage: “APOE”, “CD68”, “TREM2”; Classical Monocytes: “S100A9”, “S100A12”, “MARC1”, “FCN1”; DC cells: “CCR7”, “LAMP3”, “WFDC21P”, “BIRC3”; B cells: “CD79A”; T cells: “CD3D”, “CD3E”, “CCL5”; Fibroblasts: “PDGFRA”, “COL1A1”, “COL1A2”, “DCN”; Endothelials: “VWF”, “PECAM1”, “CLDN5”, “PLVAP”, “SPARCL1”; Smooth muscle: “ACTA2”, “MYL9”; Mast cells: “CPA3”, “TPSAB1”, “TPSB2”. The markers were cross-referenced with several public databases, including PanglaoDB (panglaodb.se), CellTypist (www.celltypist.org), and CellMarker (xteam.xbio.top/CellMarker).

For the KEGG analysis part, we excluded the cells with zero AKR1C3 expression both from cancer cells and proliferating cancer cells, resulting in a total of 6065 cells. From this pool, we separately selected 5% of the highest/lowest AKR1C3-expressing cells, generating 304 cells in each group, to perform the differential gene expression analysis. Genes with a P-value higher than 0.05 were excluded. Subsequently, the remaining genes were ranked based on their absolute log2 fold change. The top 1000 genes were selected for the KEGG analysis. The scRNA-seq dataset has been uploaded to https://www.synapse.org/, Project SynID: syn64768750.

### NADPH-Glo assay

The NADPH-Glo™ assay kit (Promega, #G9081) was used for the assay. A total of 8000 EAC cells were seeded in a white-walled 96-well plate and incubated overnight. The assay was then performed according to the manufacturer’s protocol, with triplicate for each group.

### Seahorse XF cell mito stress test and glycolysis stress test

Seahorse XF cell mito stress test Kit (Agilent Technology, #103015-100) and Glycolysis Stress Test Kit (Agilent Technology, 103020-100) were used for the assay. A total of 12000 EAC cells were seeded in the 96-well plates, the treatment groups received 6–8 Gy irradiation. The assay was then performed 24 h after irradiation according to the manufacturer’s protocol, with at least five replicates for each group.

### Co-immunoprecipitation (co-IP)

Cells were harvested and lysed with the cell lysis buffer (Cell Signaling #9803) supplemented with 1 mM PMSF on ice for 15 min, and then centrifuged at 13,000 rpm for 10 min at 4 °C. The supernatants were collected and incubated with IP-specific antibodies on a rotator at 4 °C overnight, then incubated with protein A/G beads (Dynabeads, Invitrogen) with rotation for 30 min at room temperature on the second day. The beads were washed three times with lysis buffer and then mixed with loading buffer, cooked 10 min at 70 °C, followed by the western blot assay. The antibodies used are showed in the Supplementary Table S1.

### Proteomics sample preparation and data analysis

Samples were prepared according to the protocols provided by the Proteomics Core Facility Cologne (https://proteomics.cecad-labs.uni-koeln.de/protocols). Briefly, the OE33 AKR1C3 cell line and SKGT-4 cell line underwent IP prior to mass spectrometry. Protein concentrations were determined using the Pierce™ BCA Protein Assay Kit (#23225, Thermo Fisher), and 50 µg protein of each sample was then diluted with 8 M urea/50 mM triethylammonium bicarbonate (TEAB) buffer. Subsequently, samples were incubated with 5 mM dithiothreitol (DTT) for 1 h, followed by 40 mM chloroacetamide (CAA) incubation 30 min, avoiding light exposure. Protein samples were then digested with Lysyl endopeptidase (Lys-C) at a ratio of 1/75 for 4 h and then diluted with 50 mM TEAB to achieve a final urea concentration below 2 M. Afterwards, a last digestion step with trypsin at a 1/75 enzyme-to-substrate ratio was performed overnight. Enzymatic digestion was stopped by acidifying the samples with formic acid to a final concentration of 1%, and samples were further purified using SDB-RP StageTips before measurement. The analysis was performed by LC-MS/MS on a Q-Exactive Plus mass spectrometer coupled to an Easy nanoLC 1000 system. The raw data were processed using MaxQuant^48^ with standard settings, and label-free quantification was activated.

### Cell viability assay

Four thousand OE33 cells and 7000 SKGT-4 cells were seeded each well in 96-well plates. Each group had at least triplicates. The medium was changed in the next day, the increasing concentrations of the related agents were conducted in the assigned groups. DMSO was used for the control group. After 48 or 72 h incubation, the supernatant was discarded, followed by 4 h incubation of MTT solution (Biomol #21795). Subsequently, supernatant was changed by isoproponal. After 30 min incubation at room temperature, absorbance at 570 nm was measured, and the collected data were subjected to analysis. Data analysis was performed using GraphPad Prism. Each assay was conducted in triplicate. GraphPad Prism was used for data analysis. Biological triplicates were performed in all cases.

### Flow cytometry analysis

EAC cells were seeded in the 12-well plates overnight. The second day, cells were treated with IR (6 Gy, 24–72 h) or erastin (1 µM for OE33 and 2 µM for SKGT-4, 48 or 72 h). Cells were harvested and stained with Annexin V (1.25 µL reagent in 50 µL binding buffer), 1 μM C11-Bodipy and DAPI (1:4000) for 15 min at 37 °C. Cells were then washed twice with PBS. Experiments were performed on Attune NxT Flow Cytometer and MA900FP Cell Sorter (Sony). Data analysis was done with FlowJo software (Tree Star, Ashland, USA). The fold change of the median fluorescence intensity (MFI) was calculated as: $\frac{{\rm{MFI\; of\; the\; erastin\; group}}}{{\rm{MFI\; of\; the\; corresponding\; DMSO\; group}}}$.

### Transmission electron microscopy (TEM)

Samples were prepared according to the protocols from Imaging Facility (CECAD, University of Cologne) with materials provided. Prior to cell seeding, three Aclar foils were placed into each well of a 12-well plate. The foils were then sterilized using 70% ethanol and exposed to UV light for 30 min. Subsequently, 300,000 cells were seeded into each well overnight. On the second day, the cells were treated with 2.5 Gy ionizing radiation. Twenty-four hours after treatment, the cells were fixed in 2% glutaraldehyde, 2.5% sucrose, 3 mM CaCl_2_ in 100 mM HEPES pH 7.4 for 30 min at room temperature and then 30 min at 4 °C. 3× washed with 0.1 M sodium cacodylate buffer, then incubated in 1% osmiumtetroxide, 1.25% sucrose, 1% potassium ferricyanide in 0.1 M cacodylate buffer for 1 h at 4 °C. Then washed 3× with 0.1 M cacodylate buffer. Through an row of ethanol 50–100%, a mixture ethanol/EPON and the tissue was then embedded in EPON for 72 h at 62 °C. After the embedding, the tissue was cut in 70 nm sections on the ultramicrotome (UC6, Leica) on a grid and contrasted with 1.5% uranylacetate aqueous solution 15 min at 37 °C. Washed five times in water and then incubated 4 min in lead citrate. Washed 5 times in water and dried on filter paper. Images were acquired with a transmission electron microscope (JEM 2100 Plus, JEOL), a OneView 4 K camera (Gatan) with DigitalMicrograph software at 80 KV at room temperature. Imaging analysis was performed by ImageJ.

### Patient-derived organoids (PDOs) experiments

Establishment and maintenance of EAC PDOs were described in our previous study [38]. Triplicates of single-cell digested PDOs were seeded in 48-well plates. Medium was changed on the fourth day, marked as Day 0 (D0). On the second day, which was D1, AKR1C3 inhibitor—medroxyprogesterone acetate (MPA) was used for treatment at a concentration of 20 µM. DMSO was used for the control group. On D2, two groups of PDOs were treated with 6 Gy irradiation. Images from the same sights were recorded continuously on D0–D6 but not D1 on an ECHO microscope (Model: REB-01-D). PDOs size was measured by ECHO Pro application. PDOs viability was measured by MTT assay on the last day. PDOs survival data was recorded under the microscope on D3 and the last day.

### Public data availability

The public database GEO (https://www.ncbi.nlm.nih.gov/geoprofiles/) was used for gene expression analysis in tumor and normal adjacent tissues. The public database TCGA (http://cancergenome.nih.gov/) and ferroptosis database FerrDb2 (http://www.zhounan.org/ferrdb/current/) were used to analyze prognosis and ferroptosis-related genes in EAC. Survival analysis was performed using the Kaplan–Meier method and the difference was tested with the log-rank test. A *P*-value < 0.05 was considered statistically significant.

### Statistical analysis

Statistical analysis was completed by GraphPad Prism 9. The Kaplan–Meier method was used to calculate the overall survival. Data was presented as mean ± SD, *P < 0.05, **P < 0.01, ***P < 0.001, ns: non-significant, *P* > 0.05. Statistical comparisons between groups were conducted using unpaired or paired Student’s t-test, or one-way analysis of variance (ANOVA) as appropriate for the specific experimental design and comparison being made.

## Results

### Establishment and validation of the radioresistant EAC cell line model

**Fig. 1:**
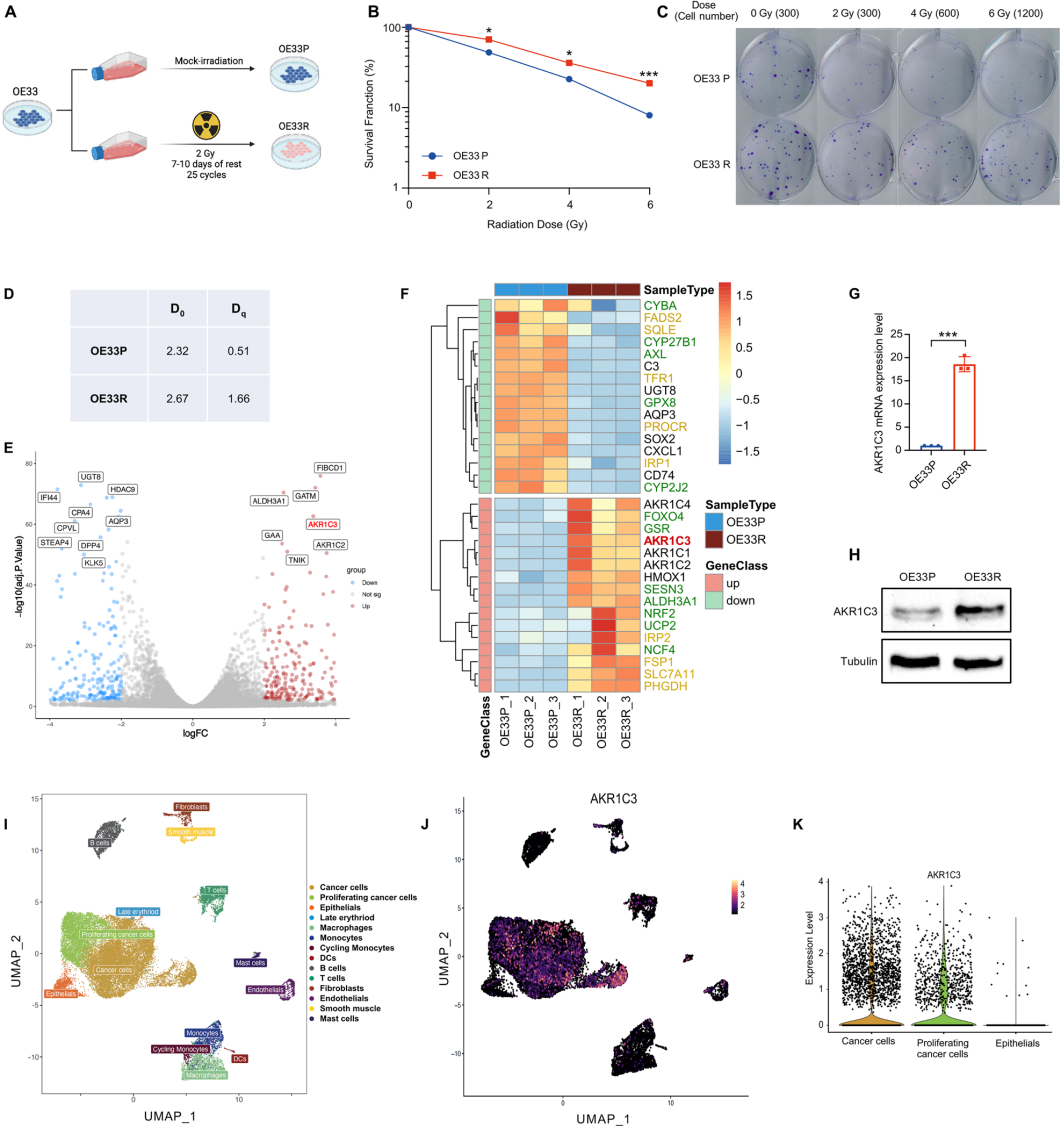
Establishment and validation of the radioresistant EAC cell line model. **A** Schematic diagram of the radioresistant model establishment. **B**, **C** Colony formation assay was performed to validate the survival curves of OE33P and OE33R. **D** The single-hit multi-target model was applied to this model. D_0_ and D_q_ values were calculated. D_0_ is the “mean lethal dose”, the dose on the straight-line portion of the survival curve to decrease the survival to 37%. D_q_ is the quasi-threshold dose, is the width of the “shoulder,” and correlates with repair capacity. **E** The volcano map exhibited the differentially expressed genes between OE33P and OE33R by the result of RNA-seq data. |logFC| > 2, log10 (adj.P.Value) > 2. **F** The major differentially expressed genes were listed in the heatmap. Marked in green: redox-related genes, marked in yellow: ferroptosis-related genes. **G** AKR1C3 mRNA relative expression level in OE33P and OE33R was measured by qRT-PCR analysis. **H** AKR1C3 protein expression level in OE33P and OE33R was validated by Western blot. Means ± SD, N = 3. **I**, **J** UMAP presented the different clusters with annotations and AKR1C3 expression in different cell types. **K** Violin plot showed the AKR1C3 expression level in cancer cells, proliferating cancer cells, and normal epithelial cells. Statistical comparisons were made using a paired two-tailed Student’s t-test; *P < 0.05, **P < 0.01, ***P < 0.001.

To deeply explore the intricate mechanisms of radioresistance in EAC, we have established a radioresistant model using the EAC cell line – OE33. The setup of the radioresistant model referred to Prof. Maher [39]. OE33 cells were initially subcultured into two T75 flasks, labeled as OE33P (the parental cell line) and OE33R (the radioresistant cell line). OE33R was exposed to 2 Gy of gamma-ray irradiation for 25 cycles (Fig. 1A). To validate the radioresistant model in vitro, we conducted a colony formation assay and found that OE33R exhibited markedly higher survival fractions compared to OE33P at irradiation doses of 2, 4, and 6 Gy (Fig. 1B, C). The single-hit multi-target model was applied to this model, showing that the D_0_ and D_q_ values of OE33R were notably higher than the parental cell line OE33P (Fig. 1D). We further conducted RNA-seq analysis on OE33P and OE33R (Supplementary RNA-seq). The sequencing data revealed a significant upregulation of AKR1C family genes (AKR1C1, AKR1C2, AKR1C3, and AKR1C4) in OE33R compared to OE33P (Fig. 1E, F). Among these, AKR1C3 had the highest expression level, while the expression of AKR1C4 was much lower than the other three genes (Table S2). Further analysis in the TCGA esophageal cancer cohort showed that AKR1C1 and AKR1C2 are downregulated in tumor tissues compared to normal tissues, and no significant difference was observed for AKR1C4. Only AKR1C3 exhibited a consistent result with our RNA-seq data (Fig. S1A–D). Therefore, we focused on AKR1C3 for further investigation. Subsequently, we validated the expression of AKR1C3 in OE33P and OE33R by qRT-PCR and Western blot, both of which confirmed a relatively higher expression level of AKR1C3 in OE33R (Fig. 1G, H). Additionally, scRNA-seq analysis was conducted on tumor tissues from four treatment-naïve EAC patients. UMAP and dotplot analyses revealed distinct cell clusters and their annotations (Figs. 1I and S1E). AKR1C3 expression was mainly observed in (proliferating) cancer cells, while being significantly low in normal epithelial cells, consistent with findings from GEO and TCGA data (Figs. 1J, K and S1F–I). These results suggested that AKR1C3 might be closely associated with radioresistance in EAC cells.

### AKR1C3 is associated with therapeutic response and prognosis of EAC patients

**Fig. 2:**
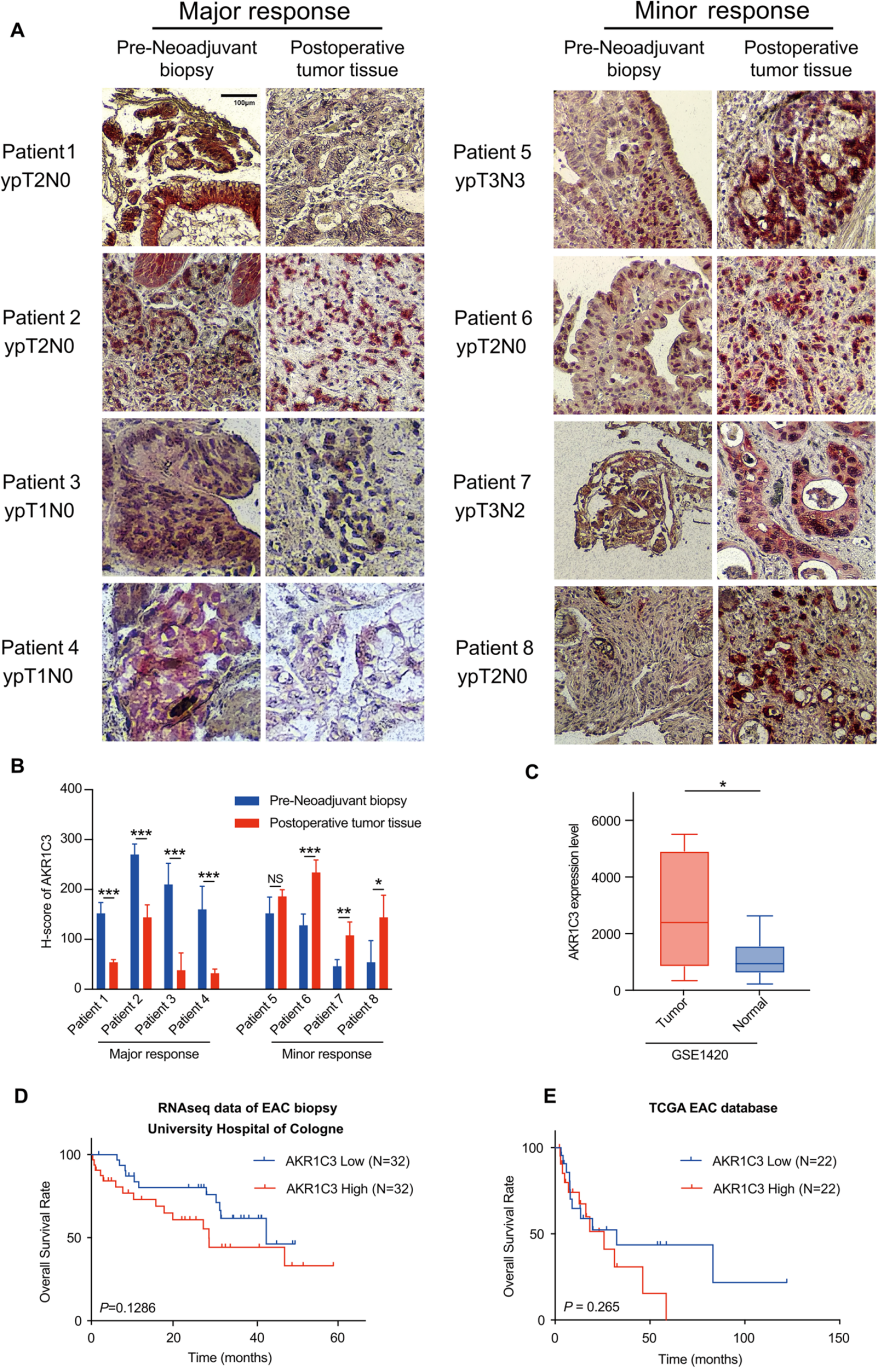
AKR1C3 affects therapeutic response and prognosis of EAC patients. **A** AKR1C3 expression levels of eight paired samples were assessed by IHC staining, all of which were diagnosed as EAC at Department of Pathology, University Hospital of Cologne. **B** The H-score of AKR1C3 was calculated as Intensity(0–3) × Area (0–100%), with five sights recorded for each sample. **C** Data from the GSE1420 dataset showed AKR1C3 expression level is higher in esophageal cancer tissues compared to the matched normal tissues. **D**, **E** Kaplan–Meier survival curves displayed the differences in overall survival rate between the AKR1C3 high (highest 25% patients) and low (lowest 25% patients) expression groups in the cohorts from University Hospital of Cologne (**D**) and TCGA (**E**). Statistical comparisons were made using a paired two-tailed Student’s t-test; *P < 0.05, **P < 0.01, ***P < 0.001.

To investigate whether AKR1C3 could influence the therapeutic response of EAC patients, we collected eight paired samples from patients who underwent CROSS treatment at the University Hospital of Cologne. Among them, four patients had major responses, and the remaining four showed minor responses. Pre-neoadjuvant biopsies were obtained from endoscopic specimens prior to any neoadjuvant treatment, whereas postoperative tumor tissues were collected after surgery from patients who had completed CROSS treatment. IHC staining revealed a noticeable downregulation of AKR1C3 levels after neoadjuvant therapy in the major response group, whereas it exhibited higher expression levels after neoadjuvant therapy in the minor response group (Fig. 2A, B). From the TCGA database, we found AKR1C3 was upregulated in tumor tissues compared to the adjacent normal tissues in esophageal cancer patients (Fig. S1C). Additionally, we further explored the public database GEO and found AKR1C3 was also upregulated in the tumor tissues compared to the adjacent normal tissues in EAC patients (Figs. 2C and S1F–I). Subsequently, we analyzed RNA-seq date from 128 EAC biopsies obtained from the patients at University Hospital of Cologne (manuscript in preparation). The AKR1C3 high group (highest 25% patients, n = 32) exhibited a better prognostic trend than the AKR1C3 low group (lowest 25% patients, n = 32) (Fig. 2D). At the same time, analysis of TCGA EAC data revealed a similar trend in overall survival rates when comparing the AKR1C3 high group (highest 25% patients, n = 22) with the AKR1C3 low group (lowest 25% patients, n = 22), consisitent with our own cohort (Fig. 2E). The combination of these findings with previous results in radioresistant EAC cells argued for a strong correlation between AKR1C3 and radioresistance in EAC.

### AKR1C3 could enhance the radioresistance in EAC cells

**Fig. 3:**
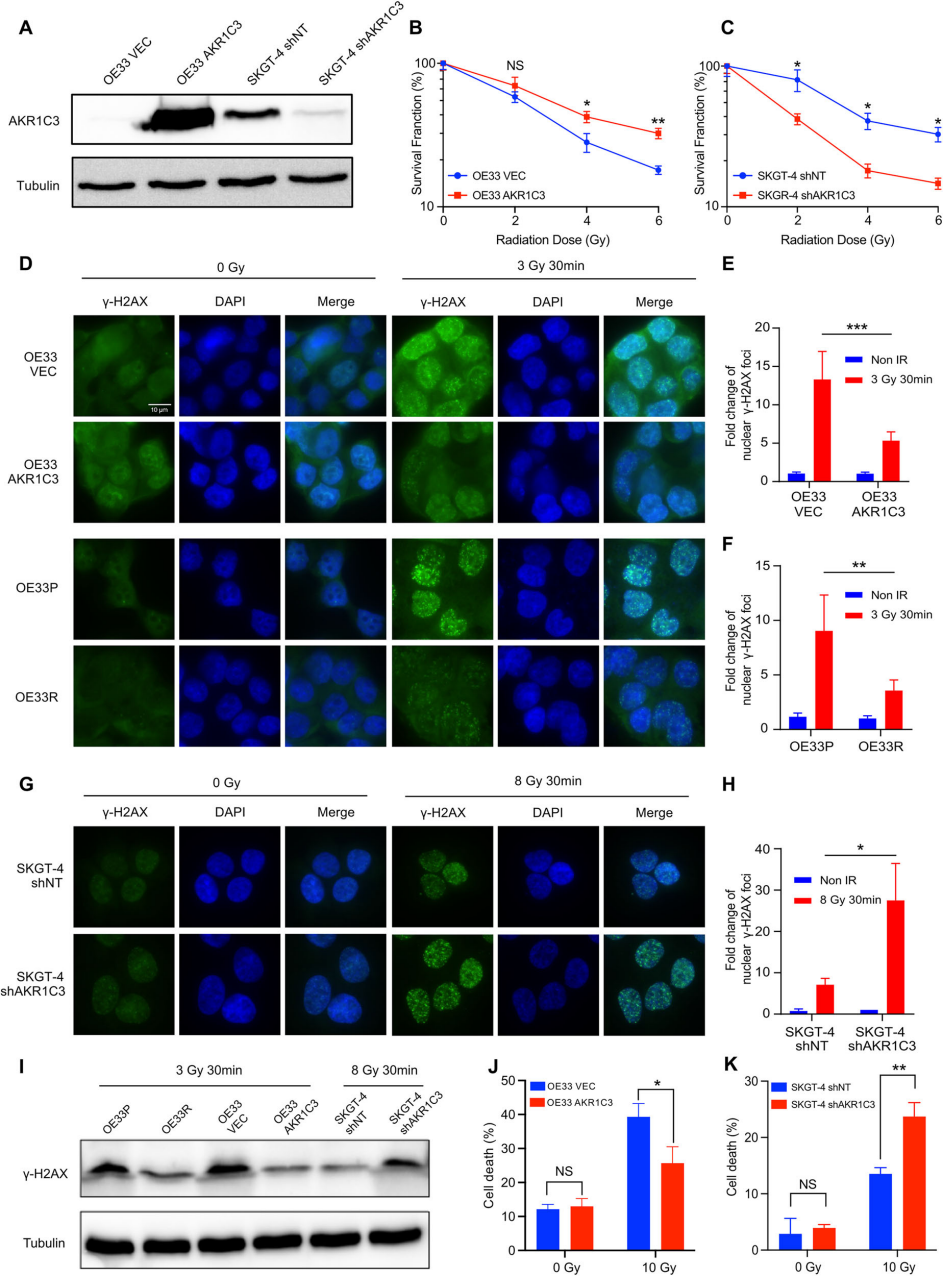
AKR1C3 could enhance the radioresistance in EAC cells. **A** Validation of AKR1C3 overexpression in OE33 and AKR1C3 knockdown in SKGT-4 was performed by western blot. **B**, **C** Survival fraction after 0–6 Gy irradiation in OE33 VEC/OE33 AKR1C3 and SKGT-4 shNT/SKGT-4 shAKR1C3. Cells were treated with irradiation: 3 Gy for OE33 (**D**) and 8 Gy for SKGT-4 (**G**). The fold change of the nuclear γ-H2AX foci was presented in the bar charts (**E**, **F**, **H**). **I** Validation of γ-H2AX protein levels in OE33P/R, OE33 VEC/AKR1C3, and SKGT-4 shNT/shAKR1C3 was performed by western blot. Cells were treated with irradiation: 3 Gy for OE33 and 8 Gy for SKGT-4. **J**, **K** Cells were treated with 0 or 10 Gy irradiation. Flow cytometry was performed 72 h after treatment. Bar charts showed the percentage of dead cells before or after irradiation in OE33 VEC/AKR1C3 and SKGT-4 shNT/shAKR1C3 by Annexin V and DAPI staining. Means ± SD, N = 3. Statistical comparisons were made using a paired two-tailed Student’s t-test; *P < 0.05, **P < 0.01, ***P < 0.001.

To better understand the mechanism of AKR1C3 and radioresistance in EAC cells, we chose OE33 VEC (control)/OE33 AKR1C3 (overexpressing) as the overexpressing model, and SKGT-4 shNT (control)/SKGT-4 shAKR1C3 (knockdown) as the knockdown model for further experiments. We performed Western blot to validate the transfection efficacy, which confirmed successful knockdown and overexpression effects (Fig. 3A). In the subsequent colony formation assay, OE33 AKR1C3 had a higher survival rate than OE33 VEC at 4 Gy and 6 Gy (Figs. 3B and S2A), while SKGT-4 shAKR1C3 had a lower survival rate than SKGT-4 shNT at 2 Gy, 4 Gy, and 6 Gy (Figs. 3C and S2B).

Subsequently, immunofluorescence and western blot of γH2AX was performed to check whether AKR1C3 could influence the DNA damage after irradiation. γH2AX serves as a sensitive molecular marker of DNA damage and repair, widely applied for cancer research [40, 41]. Cells were exposed to irradiation doses ranging from 3 to 8 Gy, followed by fixation after 30 min. OE33 AKR1C3 and OE33R presented lower fold change of nuclear γH2AX foci and lower protein levels than OE33 VEC and OE33P (Fig. 3D–F, I), indicating less DNA damage after irradiation. Similarly, SKGT-4 shAKR1C3 showed a higher fold change of nuclear γH2AX foci and a higher protein level than SKGT-4 shNT after irradiation (Fig. 3G–I), suggesting more DNA damage after irradiation for AKR1C3 knockdown cells.

Comet assay, also known as the single-cell gel electrophoresis assay, is another quick and sensitive technique for detecting DNA damage at the single-cell level [42]. In this assay, damaged DNA migrates through an electrophoresis gel, creating a “tail” resembling a passing comet. Longer and thicker tails indicate more severe DNA damage. The percentage of Tail DNA had no significant difference between OE33 VEC/OE33 AKR1C3 or OE33P/OE33R before irradiation, however, OE33 AKR1C3 and OE33R exhibited shorter DNA tails than OE33 VEC and OE33P after 6 Gy irradiation (Fig. S2C, D, F, G), which means OE33 AKR1C3 and OE33R had less DNA damage after irradiation compared to OE33 VEC and OE33P. Additionally, SKGT-4 shAKR1C3 presented significantly longer DNA tails than SKGT-4 shNT after 10 Gy irradiation (Fig. S2E, H). However, we observed that SKGT-4 shAKR1C3 also had a higher percentage of Tail DNA before irradiation, which may be related to the knockdown effect. Besides, Annexin V and DAPI were used for staining in flow cytometry to identify the dead cells. The results showed no significant difference between OE33 VEC/OE33 AKR1C3, nor SKGT-4 shNT/SKGT-4 shAKR1C3 before irradiation. While 72 h after 10 Gy irradiation, OE33 AKR1C3 had remarkably fewer dead cells than OE33 VEC (Figs. 3J and S2I). On the contrary, 72 h after 10 Gy irradiation, SKGT-4 shAKR1C3 had more dead cells than SKGT-4 shNT (Figs. 3K and S2J). Taken together, these findings suggested that high expression of AKR1C3 could reduce DNA damage and enhance radioresistance in EAC cells.

### AKR1C3 is associated with ferroptosis

**Fig. 4:**
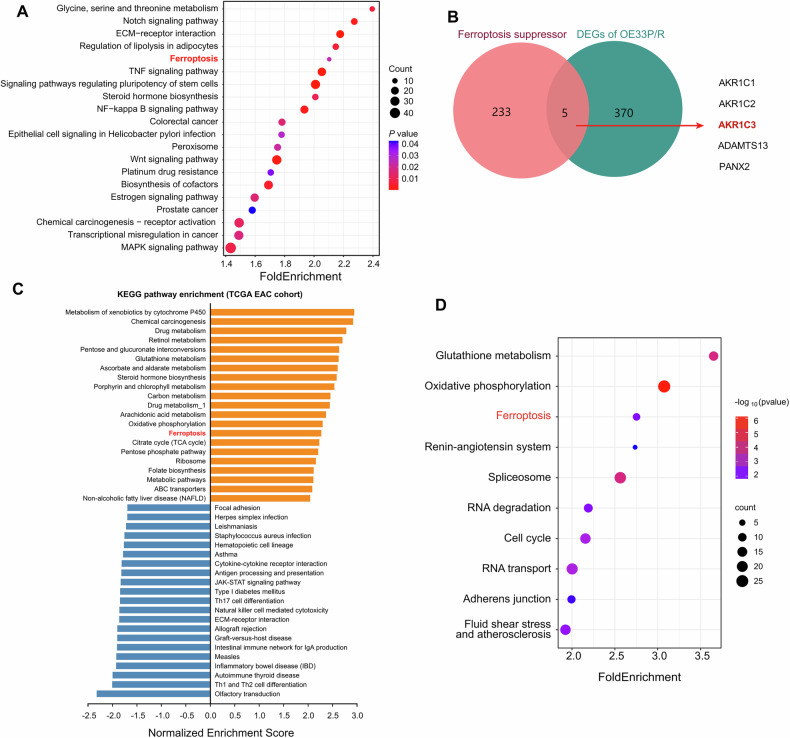
AKR1C3 is associated with ferroptosis. **A** KEGG pathways ranked by fold enrichment were analyzed based on the DEGs of OE33P/OE33R. **B** Venn diagram analysis illustrated the five genes overlapped between 238 ferroptosis suppressors and 375 DEGs of OE33P/R. **C** KEGG pathways ranked by fold enrichment were analyzed based on the DEGs of the AKR1C3 high/low group from the TCGA EAC cohort. **D** KEGG pathways ranked by fold enrichment were analyzed based on the DEGs of the AKR1C3 high/low group from scRNA-seq.

To further explore the mechanism linking AKR1C3 and radioresistance, we performed KEGG pathway analysis based on our RNA-seq data of OE33P/R. Our analysis revealed enrichment of several classic pathways, including Notch, NF-kappa B, and Wnt signaling pathways. Notably, among the differentially expressed genes (DEGs) of OE33P/R, 7 out of 41 ferroptosis-associated genes were enriched (Fig. 4A). This finding piqued our interest, particularly in light of our previous research indicating that AKR1C3 can regulate GSH levels—a critical antioxidant involved in detoxifying ROS in cells [20]—which also plays a pivotal role in ferroptosis. Subsequently, we intersected the 238 ferroptosis suppressors from FerrDb2 database with the DEGs of OE33P/R, five genes (AKR1C3, AKR1C2, AKR1C1, ADAMTS13 and PANX2) were identiified (Fig. 4B). Subsequently, we filtered TCGA EAC cohort, excluded the patients who received radiotherapy, and ranked the patients by AKR1C3 expression level, half patients were categorized into the AKR1C3 high group, and the other half into the AKR1C3 low group. DEGs were extracted from these two groups. Through the following KEGG analysis, we found ferroptosis was also enriched in AKR1C3 high group of TCGA EAC cohort (Fig. 4C). Interestingly, some pathways which were associated with AKR1C3 or ferroptosis were also enriched, such as retinol metabolism, glutathione metabolism, and steroid hormone biosynthesis. Additionally, we filtered cancer cells and proliferating cancer cells from our scRNA-seq data, and performed a differential gene expression analysis between AKR1C3 high and low groups. Subsequent KEGG analysis results showed significant enrichment of ferroptosis as well as glutathione metabolism pathways (Fig. 4D). Although ferroptosis was not the most significantly enriched pathway, it was consistently enriched across all datasets. Moreover, many of the other enriched pathways were closely related to ferroptosis, such as peroxisome function, glutathione metabolism, and oxidative phosphorylation. Overall, these findings suggest that ferroptosis may play a critical role in AKR1C3-mediated radioresistance, potentially through its interplay with these metabolic and redox-related pathways.

### AKR1C3 regulates redox homeostasis and inhibits ferroptosis in EAC cells

**Fig. 5:**
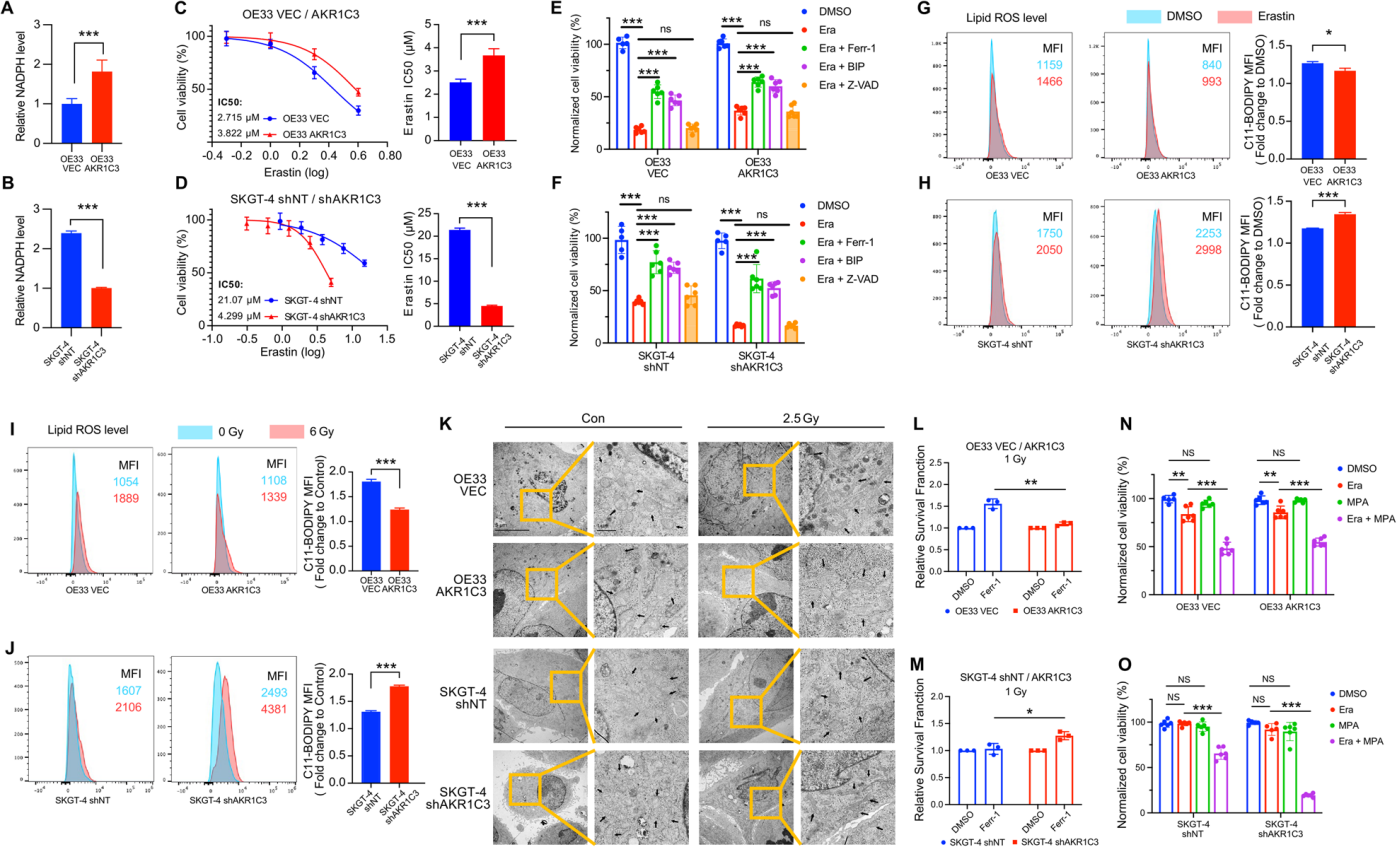
AKR1C3 regulates redox homeostasis and inhibits ferroptosis in EAC cells. **A**, **B** The luminescence of NADPH was measured by the luminometer 30 min after incubation with NADPH-Glo™ Detection Reagent. **C**, **D** OE33 VEC/AKR1C3 and SKGT-4 shNT/shAKR1C3 cells were treated with 0.5–4 µM and 1.25–20 µM erastin, respectively for 72 h. The relative cell viability was measured by the MTT assay. **E**, **F** OE33 VEC/AKR1C3 and SKGT-4 shNT/shAKR1C3 cells were treated with erastin (3 µM and 8 µM, respectively) for 72 h, with 10 µM Ferr-1 for 72 h, with 50 µM BIP for 72 h, or with Z-VAD-FMK 5 µM for 72 h. The relative cell viability was measured by the MTT assay. Data was normalized with the DMSO groups. **G**, **H** Lipid peroxidation level was detected by C11-Bodipy staining on flow cytometry. The concentration of C11-Bodipy for staining was 1 µM. OE33 and SKGT-4 cells were treated with 1 µM and 2 µM erastin for 48 h, respectively. Bar graphs showing erastin-induced relative fold change of lipid peroxidation levels. **I**, **J** OE33 VEC/AKR1C3 and SKGT-4 shNT/shAKR1C3 cells were treated with 6 Gy. After 48 h, lipid peroxidation level was detected by C11-Bodipy staining on flow cytometry. The concentration of C11-Bodipy for staining was 1 µM. Bar graphs showing irradiation-induced relative fold change of lipid peroxidation levels. **K** TEM images of OE33 VEC/AKR1C3 and SKGT-4 shNT/shAKR1C3 before and after radiotherapy (2.5 Gy, fixation after 24 h). Black arrow: mitochondria. A minimum of five cells in each group were examined. **L**, **M** 500 cells were seeded in the 6-well plated, 1 Gy irradiation on the second day, the concentration of Ferr-1 was 0.3 µM. Cells were fixed and counted after 7–12 days. Data was normalized with the DMSO groups. **N**, **O** OE33 VEC/AKR1C3 and SKGT-4 shNT/shAKR1C3 cells were treated with 1.5 µM erastin, 10 µM MPA, and combined use of erastin/MPA for 48 h. The relative cell viability was measured by the MTT assay. Data was normalized with the DMSO groups. Mean ± SD, N = 3. *P < 0.05, **P < 0.01, ***P < 0.001.

GSH, a major cellular antioxidant, helps to maintain redox homeostasis by neutralizing ROS and preventing oxidative stress and ferroptosis. In our previous study, we validated that GSH can be upregulated by the AKR1C3-AKT axis [20]. NADPH, a key material for GSH synthesis and a crucial regulator of redox homeostasis, is also essential for AKR1C3-induced redox reactions. To assess the redox status in EAC cells, we performed the NADPH-Glo™ assay. The NADPH level was found to be higher in OE33 AKR1C3 compared to OE33 VEC, and lower in SKGT-4 shAKR1C3 compared to SKGT-4 shNT (Fig. 5A, B). This suggests that AKR1C3 may promote NADPH generation and provide the essential material for GSH synthesis, thereby contributing to the cellular redox homeostasis. To further explore whether AKR1C3 could regulate ferroptosis as we previously hypothesized, erastin (a ferroptosis inducer) was used to treat EAC cell lines. We performed MTT assay to assess the sensitivity of erastin in EAC cells. IC50 of erastin was significantly higher in OE33R and OE33 AKR1C3 than in OE33P and OE33 VEC (Figs. 5C and S3A), while it was lower in SKGT-4 shAKR1C3 than in SKGT-4 shNT (Fig. 5D), indicating that AKR1C3 confers erastin resistance in EAC cells. Subsequently, we found that the cell death induced by erastin could be partially rescued by the antioxidant Ferrostatin-1 (Ferr-1) and the iron chelating agent 2,2’-bipyridine (BIP), but not by the apoptosis inhibitor Z-VAD-FMK (Fig. 5E, F). In addition, flow cytometry assay after erastin treatment showed that the median fluorescence intensity (MFI) of C11-Bodipy, an indicator for lipid ROS level, in OE33R and OE33 AKR1C3 was lower than in OE33P and OE33 AKR1C3 (Figs. 5G and S3B), while the fold change of MFI in SKGT-4 shAKR1C3 was higher than in SKGT-4 shNT (Fig. 5H), suggesting AKR1C3 could prevent EAC cells from lipid peroxidation, thereby inhibiting ferroptosis upon erastin treatment. We also detected the lipid peroxidation levels after radiotherapy, which presented a similar trend to erastin-induced lipid peroxidation. After radiotherapy, OE33R and OE33 AKR1C3 exhibited lower lipid ROS levels than in OE33P and OE33 AKR1C3 (Figs. 5I and S3C), while the lipid ROS level in SKGT-4 shAKR1C3 was higher than in SKGT-4 shNT (Fig. 5J).

Subsequently, TEM was performed to observe the morphological changes of mitochondria in EAC cells. We observed the distinctive morphological changes in OE33P/OE33 VEC and SKGT-4 shAKR1C3 after radiotherapy, which appeared to have a more condensed membrane and more elongated mitochondrial characteristics (Figs. 5K and S3D), consistent with the typical ferroptosis morphology [43]. While OE33R exhibited these morphological changes prior to radiotherapy, possibly due to prolonged low-dose radiotherapy (Fig. S3D), and remained unchanged post-radiotherapy, suggesting resistance to this stimulus. Moreover, tetramethyl rhodamine ethyl ester (TMRE) staining, a cell-permeant fluorescent dye used for labeling active mitochondria, revealed that MFI was upregulated in OE33 AKR1C3 and downregulated in SKGT-4 shAKR1C3 post-radiotherapy (Fig. S3E, F), indicating that AKR1C3 can regulate mitochondrial activity in EAC cells. To further explore whether AKR1C3 could affect mitochondrial metabolism, we performed Seahorse XF cell mito stress test and Seahorse XF glycolytic rate assay to measure oxygen consumption rate (OCR) and extracellular acidification rate (ECAR), respectively. OE33 AKR1C3 showed a more significant upregulation of OCR and ECAR after radiotherapy than OE33 VEC (Fig. S3G, H). SKGT-4 shNT also showed a higher OCR and ECAR upregulation than SKGT-4 shAKR1C3 after radiotherapy (Fig. S3I, J). These results suggest that AKR1C3 plays an important role in regulating mitochondrial metabolism in EAC cells.

As a next step, we tried to ascertain whether AKR1C3-induced radioresistance was attributable to ferroptosis inhibition. We performed the colony formation assay, treating cells with 1 Gy irradiation followed by the addition of either Ferr-1 or DMSO the next day. The relative survival fraction presented that the rescue effect of Ferr-1 in OE33P, OE33 VEC, and SKGT-4 shAKR1C3 was more significant than in OE33R, OE33 AKR1C3, and SKGT-4 shNT (Figs. 5L, M and S3K). The results indicated Ferr-1 could partially rescue the cell death induced by radiotherapy, and this effect was more pronounced in cells with low AKR1C3 expression.

Medroxyprogesterone acetate (MPA), a hormonal medication of the progestin type, has been reported as an effective inhibitor to suppress the enzyme activity of AKR1C3 [44]. Initially, we evaluated the cytotoxicity of MPA by MTT assay, we found MPA showed low cytotoxicity on cells at concentrations up to 15 µM (Fig. S3L–N). Subsequently, we analyzed the AKR1C3 expression level by Western blot following 1–10 µM MPA treatment for 72 h, results revealed no significant alteration in AKR1C3 protein levels (Fig. S3O). As we know, AKR1C3 is a NADPH-dependent enzyme. To evaluate the inhibitory effect of MPA on AKR1C3, we performed an NADPH assay. EAC cells were cultured in glucose/glutamine-free medium to block NADPH synthesis, and 10 µM MPA was added for 2 h. We observed that NADPH levels were upregulated in both OE33 and SKGT-4 cell lines, with a more significant increase in SKGT-4 (Fig. S3P). These results suggested that MPA effectively inhibits the enzymatic activity of AKR1C3 in EAC cells. Then, we performed MTT assay to check the cell viability after treatment with erastin alone, MPA alone, or erastin plus MPA. Interestingly, we found that the combined use of erastin and MPA resulted in a significant decrease in cell viability than using erastin alone (Figs. 5N, O and S3Q), suggesting the inhibition of AKR1C3 enzyme activity could sensitize EAC cells to erastin.

In summary, these findings validated that AKR1C3 could mediate ferroptosis in EAC cells.

### AKR1C3 inhibits ferroptosis by suppressing TRIM21-mediated ubiquitination of HSPA5

**Fig. 6:**
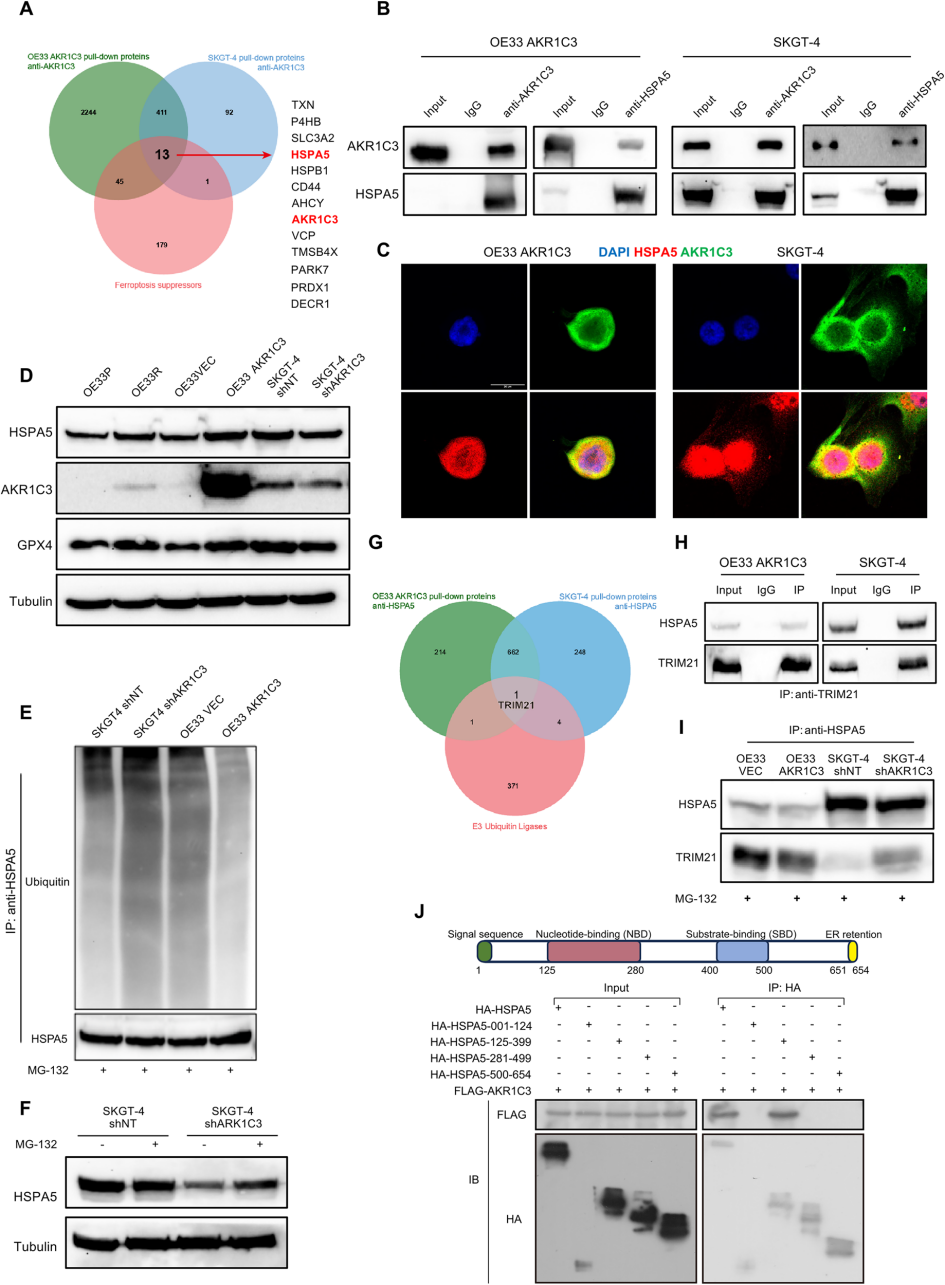
AKR1C3 inhibits ferroptosis by suppressing TRIM21-mediated ubiquitination of HSPA5. **A** Proteomic analysis was conducted on the immunoprecipitated products from anti-AKR1C3 co-IPs of OE33 AKR1C3 and SKGT-4. Venn diagram exhibited the integration of proteomic results with 234 ferroptosis suppressors. Totally 13 enriched genes were listed. **B** Co-IP assay results presented the interactions between AKR1C3 and HSPA5 in EAC cells. **C** Immunofluorescence assay results showed the co-localization of AKR1C3 with HSPA5 in EAC cells. **D** Western blot results exhibited the protein expression of HSPA5, AKR1C3, and GPX4 in different EAC cell lines. **E** Western blot results exhibited ubiquitin level of HSPA5 in different EAC cell lines. **F** Western blot results showed the protein level of HSPA5 with or without MG-132 treatment in SKGT-4 shNT/shAKR1C3 cell lines. **G** Proteomic analysis was conducted on the immunoprecipitated products from anti-HSPA5 co-IPs of OE33 AKR1C3 and SKGT-4. Venn diagram exhibited the integration of proteomic results with 377 E3 ubiquitin ligases. TRIM21 was enriched. **H** Co-IP assay results presented the interactions between TRIM21 and HSPA5 in EAC cells. **I** Co-IP assay results presented the different strengths of interactions between TRIM21 and HSPA5 in EAC cells. **J** Analyses of HSPA5 binding domains with AKR1C3. Four truncated forms of HA-HSPA5 were individually co-transfected with Flag-tagged AKR1C3 into HEK293T cells, and co-IP was performed with the anti-HA antibody.

To investigate the role of AKR1C3 in regulating ferroptosis, we performed proteomics on the immunoprecipitated products by anti-AKR1C3 co-IPs of OE33 AKR1C3 and SKGT-4, which express relatively high levels of AKR1C3. We integrated the results with ferroptosis suppressors identified from FerrDb2 database, revealing 13 candidate genes (Fig. 6A). Interestingly, HSPA5, also known as binding immunoglobulin protein (BiP) or 78 kDa glucose-regulated protein (GRP78), captured our attention (Fig. S4A). Recent studies have demonstrated that the HSPA5/GPX4 axis negatively regulates ferroptosis by stabilization of GPX4 [45–48]. We validated our proteomics results by anti-AKR1C3 and anti-HSPA5 co-IPs in OE33 AKR1C3 and SKGT-4 (Fig. 6B). Co-localization analysis by immunofluorescence exhibited ARK1C3 mainly interacted with HSPA5 in the cytoplasm (Fig. 6C). Analysis of GEO databases showed HSPA5 was upregulated in EAC tumor tissues compared to the normal adjacent tissues (Fig. S4B, C). Moreover, the protein levels of HSPA5 and GPX4 were positive related to AKR1C3 protein levels (Fig. 6D). Subsequent RNAi targeting HSPA5 in OE33 and SKGT-4 cells resulted in downregulation of GPX4 protein levels (Fig. S4D), suggesting that AKR1C3 could regulate the HSPA5/GPX4 axis. Since our RNA-seq data from OE33P/R cells did not show significant differences in HSPA5 mRNA levels, we speculated that post-transcriptional modifications might contribute to this regulation. Reviewing the literature, we found many studies implicating the ubiquitination of HSPA5, which are closely related to the development of tumors [49–51]. Hence, we accessed the ubiquitination level of HSPA5 in EAC cells. SKGT-4 shAKR1C3 showed higher ubiquitination level of HSPA5 than SKGT-4 shNT, while OE33 AKR1C3 presented lower ubiquitination level of HSPA5 compared to OE33 VEC (Fig. 6E). To investigate whether AKR1C3 affects the half-life of HSPA5 protein, cycloheximide was applied to block the protein biosynthesis. Shorter protein half-life of HSPA5 was found in OE33 VEC and SKGT-4 shAKR1C3 compared to OE33 AKR1C3 and SKGT-4 shNT (Fig. S4E). Furthermore, treatment with MG-132, a potent proteasome inhibitor, rescued HSPA5 protein levels notably in SKGT-4 shAKR1C3, but not significantly in SKGT-4 shNT (Fig. 6F). These results indicated AKR1C3 could suppress the ubiquitionation degradation of HSPA5 through proteasome-dependent pathway.

To study the in-depth mechanism of ubiquitination involved in AKR1C3 and HSPA5, we performed another round of proteomics on the immunoprecipitated products by anti-HSPA5 co-IPs of OE33 AKR1C3 and SKGT-4. The results were integrated with 377 E3 ubiquitin ligases [52], TRIM21 was the only E3 ubiquitin ligase identified (Figs. 6G and S4F). We conducted co-IPs to confirm the proteomics results (Fig. 6H, I). Interestingly, TRIM21 was less immunoprecipitated by anti-HSPA5 in OE33 AKR1C3 compared to OE33 VEC, conversely, it was more immunoprecipitated in SKGT-4 shAKR1C3 compared to SKGT-4 shNT (Fig. 6I). The protein level of TRIM21 was upregulated in SKGT-4 shAKR1C3 compared to SKGT-4 shNT, while it presented no difference between OE33 VEC/AKR1C3 (Fig. S4G). Based on these findings, we hypothesized AKR1C3 could bind to HSPA5, thereby preventing HSPA5 degradation from TRIM21-induced ubiquitination. To validate this hypothesis, we knockdowned TRIM21 in SKGT-4 shAKR1C3, the ubiquitination level was reduced (Fig. S4H, I). Besides, overexpression of TRIM21 in OE33 AKR1C3 rescued the ubiquitination level of HSPA5 (Fig. S4J, K). HSPA5 is a protein containing two structural domains: a nucleotide-binding domain and a substrate-binding domain [53]. To determine the domains for AKR1C3-HSPA5 interaction, plasmids encoding the full length of Flag-tagged AKR1C3 and 4 HA-tagged truncated mutants of HSPA5, were constructed for IP analyses. Our results indicated that AKR1C3 bound to the nucleotide-binding domain (125 - 399 aa) of HSPA5 (Fig. 6J). Our current findings indicated AKR1C3 could bind to the nucleotide-binding domain of HSPA5 and reduce the interaction between HSPA5 and TRIM21 to stabilize HSPA5/GPX4 axis, further inhibiting ferroptosis.

### Inhibition of AKR1C3 resensitizes EAC PDOs to radiotherapy

**Fig. 7:**
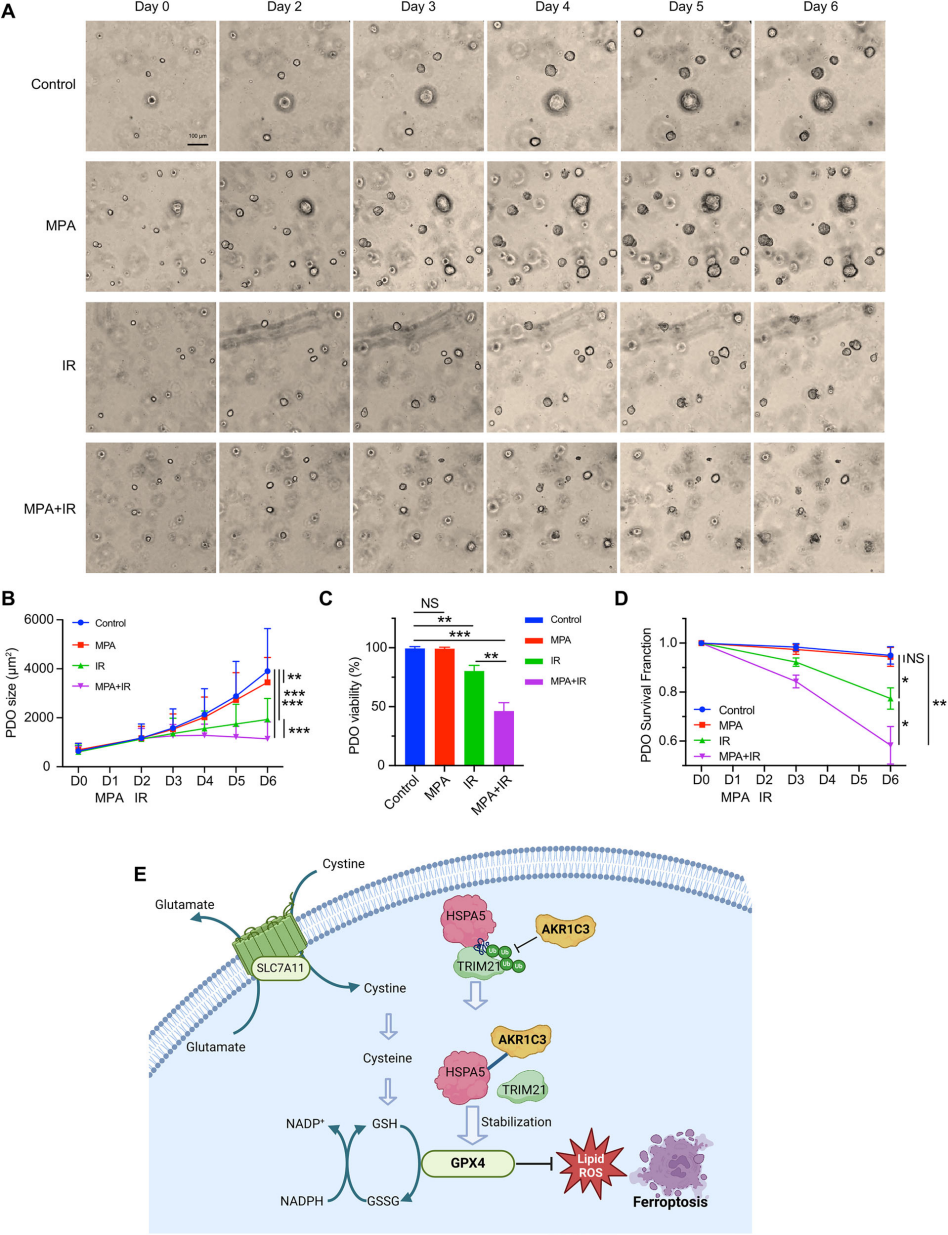
Inhibition of AKR1C3 resensitizes EAC PDOs to radiotherapy. **A** Triplicates of single-cell digested PDOs were seeded in 48-well plates. Images showed the morphological changes from D0 to D6 but not D1. **B** PDOs size presented the growth process from D0 to D6. Size was measured by ECHO Pro application. **C** PDOs viability was measured by MTT assay on the last day. **D** PDOs survival data were recorded under the microscope on D3 and the last day. Mean ± SD, N = 3. * *P* < 0.05, ** *P* < 0.01, *** *P* < 0.001. **E** Schematic diagram illustrating the mechanism of AKR1C3 inhibiting ferroptosis in EAC cells. Briefly, AKR1C3, upregulated in the radioresistant EAC cells, binds to HSPA5 and reduced the interaction between TRIM21 and HSPA5, thereby prevents HSPA5 degradation from TRIM21-induced ubiquitination. HSPA5 could futher stabilize GPX4, subsequently suppressing ferroptosis.

Having revealed AKR1C3 enhances radioresistance via inhibiting ferroptosis in EAC cells, we subsequently investigate the potential of pharmacologically targeting AKR1C3 to improve the radiotherapy response in EAC PDOs. From our established PDO cohort, we selected a PDO with high AKR1C3 expression for further experimental analysis. Triplicates of single-cell digested PDOs were seeded in 48-well plates. Medium was changed on the fourth day, marked as Day 0 (D0). On the second day (D1), AKR1C3 inhibitor MPA was applied for treatment at a concentration of 20 µM. On D2, two groups of PDOs were treated with 6 Gy irradiation. Images from the same sights were recorded continuously on D0 to D6, excluding D1 (Fig. 7A). Sole application of MPA conferred a slight influence on PDOs size, but no significant effect on viability or survival rate (Fig. 7B–D). However, when combined with radiotherapy, MPA significantly suppressed growth speed and survival rate, indicating MPA could resensitize EAC PDOs to radiotherapy (Fig. 7B–D). Overall, our findings revealed that AKR1C3 is a promising target for improving the response to radiotherapy in EAC, highlighting its critical role of inhibiting ferroptosis through the stabilization of HSPA5/GPX4 axis. Therefore, targeting AKR1C3 represents a potentially effective approach for overcoming radioresistance in EAC (Fig. 7E).

## Discussion

Despite advancements in multimodal cancer treatments, the prognosis of EAC is still dismal. Therapy resistance, notably chemoresistance and radioresistance, appears to be mainly responsible for the poor prognosis of EAC patients [54–56]. Our previous study demonstrated AKR1C3 could mediate chemoresistance via detoxification of ROS; additionally, emerging evidence suggested that AKR1C3 also enhanced radioresistance in esophageal squamous cancer cells (ESCC) [19, 57, 58]. However, specific mechanisms through which AKR1C3 confers radioresistance have not been deeply studied in EAC.

Xiong et al. [19] observed significantly upregulated levels of AKR1C3 expression in radioresistant ESCC cell lines KY170 and TE13, and validated its pivotal role in oxidative stress response. We sought to investigate whether AKR1C3 functions similarly in EAC or if distinct phenotypes and mechanisms exist in this context. Our initial step was establishing a radioresistant cell line for EAC in vitro. Among different EAC cell lines, SKGT-4 and OE19 displayed relatively low response to irradiation, whereas OE33 exhibited relatively more sensitive. Following Prof. Maher’s method and suggestion [39], we successfully established OE33 as a radioresistant EAC model. Our data confirmed that the radioresistant OE33 cell line (OE33R) exhibited higher survival fractions compared to the parental OE33 cell line (OE33P). RNA-seq data revealed significant upregulation of AKR1C3 in OE33R, consistent with findings in ESCC radioresistance models [19]. Additionally, other members of the AKR family, including AKR1C1, AKR1C2, and AKR1C4, were also upregulated in OE33R, suggesting the critical role of the AKR family in the modulation of radioresistance. Interestingly, some redox-related genes, such as GSR and NRF2 (marked in green in Fig. 1F), along with ferroptosis-related genes (marked in yellow in Fig. 1F) like SLC7A11 and FSP1, were differentially expressed between OE33P and OE33R, suggesting that redox homeostasis and ferroptosis may play critical roles in the regulation of radioresistance in EAC. Consistent with our findings in the radioresistant cell line, AKR1C3 exhibited an upregulated trend in CROSS-minor response patients from our IHC results. Furthermore, patients with high AKR1C3 expression levels presented a poorer prognosis. Given our prior discoveries and the extensive studies on AKR1C3, we decided to investigate more on AKR1C3 into unraveling novel molecular mechanisms of radioresistance.

Irradiation functions by releasing electrons, generating high-energy damage that induces DNA double-strand breaks (DSBs), while also generating ROS through water radiolysis, contributing to DNA damage [59]. Our research demonstrated that AKR1C3 could reduce IR-induced DNA damage and cell death, conferring radioresistance in EAC cells. Our previous study demonstrated that AKR1C3 could regulate intracellular ROS levels in EAC cells, which partly explained the effect of ARK1C3 on IR-induced DNA damage and cell death [20]. Nonetheless, given the numerous signaling pathways linked with ROS, further investigation is warranted to elucidate the precise mechanism of radioresistance.

Following a comprehensive analysis of (sc)RNA-seq data from both our own cohort and public databases, including EAC cell lines and tumor tissues, the pathway of ferroptosis was significantly enriched. Beyond ferroptosis, our KEGG analysis also indicated that AKR1C3 is involved in multiple metabolic pathways which are highly associated with ferroptosis, including regulation of lipolysis in adipocytes, peroxisome, glutathione metabolism, pentose phosphate pathway, and oxidative phosphorylation (Fig. 4). Scott J Dixon et al. isolated five clonal cell lines from prostate cancer cells which were strongly resistant to erastin and then performed RNA-seq for these clonal cell lines versus the parental cell line. AKR1C3, as well as AKR1C1/AKR1C2, were significantly upregulated in the RNA-seq data, suggesting AKR1C family might cause erastin-resistance by the prevention of lipid peroxidation [60]. Our study further demonstrated that AKR1C3 exhibited resistance to erastin in EAC cells, which was consistent with the results mentioned above. TEM and C11-Bodipy staining further confirmed AKR1C3’s regulation of ferroptosis in EAC cells. Additionally, TMRE and Seahorse results demonstrated AKR1C3 enhanced mitochondrial activity and metabolism. Interestingly, Wu et al. reported that AKR1C3 resulted in a higher ECAR/OCR ratio, suggesting that AKR1C3 could drive a metabolic shift from oxidative phosphorylation to glycolysis in HCC cells [16]. Our results also revealed that AKR1C3 can lead to an increase in ECAR levels following irradiation. The glycolytic intermediates, such as glucose-6-phosphate, can serve as substrates for the pentose phosphate pathway, resulting in the production of additional NADPH to help protect cells from oxidative stress [61]. Moreover, ferr-1, a ferroptosis inhibitor, was found to have a stronger capacity to rescue the cell death induced by erastin or irradiation in AKR1C3 low-expressing cells than in AKR1C3 high-expressing cells in our present study. Inhibition of AKR1C3 also sensitized cells to erastin. So far, we have demonstrated AKR1C3 could inhibit IR-induced ferroptosis. Through proteinomics, we identified an interesting protein—HSPA5, which was reported to stabilize GPX4 and inhibit ferroptosis in cancers [45]. This study focused on improving the gemcitabine sensitivity in pancreatic ductal adenocarcinoma (PDAC) by targeting ATF4/HSPA5/GPX4 axis, while the exact molecular mechanism was not deeply studied. Additionally, some studies revealed that HSPA5 could be modified and degraded by ubiquitin-mediated poly-ubiquitylation, resulting in inhibition of tumorigenesis [49, 50, 62]. To the best of our knowledge, this study is the first to report that AKR1C3 can bind to HSPA5 and reduce the interaction between HSPA5 and TRIM21, thereby protecting HSPA5 from TRIM21-induced ubiquitination degradation.

So far, numerous studies have shown AKR1C3 could serve as a potential biomarker across various cancers. Peraldo-Neia et al. reported that inhibition of AKR1C3 could suppress cell proliferation and sensitize cells towards chemotherapy in oropharynx squamous cell carcinoma (OPSCC). Additionally, AKR1C3 expression levels in 111 independent OPSCC patients were positively correlated with the poor prognosis [63]. Furthermore, other studies indicated that AKR1C3 has a potential value in the clinical diagnosis of T-cell acute lymphoblastic leukemia/lymphoma [64, 65]. In prostate cancer, AKR1C3 expression was positively correlated with the Gleason score, indicating AKR1C3 could serve as a promising biomarker for the prognosis [66]. In our current and previous studies, we are the first to validate the role of AKR1C3 in mediating chemoresistance and radioresistance in EAC cells, to show its association with CROSS treatment responses in EAC biopsies and tumor tissues. Generally, AKR1C3 acts as a promising biomarker and therapeutic target in EAC patients.

OBI-3424, a potent DNA-alkylating prodrug which is selectively activated by AKR1C3 [67], has been evaluated for the safety, pharmacodynamics, and preliminary efficacy in an acute lymphoblastic leukemia study [67] and a phase 1 clinical trial of advanced solid tumors [68], currently being investigated in multiple phase 2 clinical trials in the USA (NCT03592264). Another phase 1/2 clinical trial (NCT06245330) is currently evaluating another AKR1C3 prodrug – AST-001 [69] in subjects with advanced solid tumors. In our future research, we will emphasize targeting therapy, particularly focusing on in vivo experiments and clinical applications, translating our findings into clinical benefits for EAC patients.

This study revealed the critical role of AKR1C3 in the radioresistance of EAC. AKR1C3 can inhibit ferroptosis through stabilization of HSPA5/GPX4 axis. In the future, in vivo experiments targeting AKR1C3 might bring more pre-clincial insights for individual care of EAC patients. Subsequent clinical trials may further validate these findings and offer new therapeutic approaches in patients with EAC or other cancer entities.

## Supplementary information

Supplementary RNA-seq

Supplementary data

Original blot

## References

[1] Bray F, Laversanne M, Sung H, Ferlay J, Siegel RL, Soerjomataram I, et al. Global cancer statistics 2022: GLOBOCAN estimates of incidence and mortality worldwide for 36 cancers in 185 countries. CA Cancer J Clin. 2024;74:229–63. 38572751 10.3322/caac.21834

[2] Joseph A, Raja S, Kamath S, Jang S, Allende D, McNamara M, et al. Esophageal adenocarcinoma: a dire need for early detection and treatment. Cleve Clin J Med. 2022;89:269–79. 35500930 10.3949/ccjm.89a.21053

[3] Shapiro J, van Lanschot JJB, Hulshof M, van Hagen P, van Berge Henegouwen MI, Wijnhoven BPL, et al. Neoadjuvant chemoradiotherapy plus surgery versus surgery alone for oesophageal or junctional cancer (CROSS): long-term results of a randomised controlled trial. Lancet Oncol. 2015;16:1090–8. 26254683 10.1016/S1470-2045(15)00040-6

[4] van Laarhoven H, Verhoeven R, van Berge Henegouwen M, Mohammad NH, van Hillegersberg R, Slingerland M, et al. Real-world outcomes of the CROSS regimen in patients with resectable esophageal or gastro-esophageal junction adenocarcinoma: a nationwide cohort study in the Netherlands. EClinicalMedicine. 2025;80:103067. 39911244 10.1016/j.eclinm.2024.103067 PMC11795631

[5] Lagergren J, Lagergren P. Recent developments in esophageal adenocarcinoma. CA Cancer J Clin. 2013;63:232–48. 23818335 10.3322/caac.21185

[6] Ho ALK, Smyth EC. A global perspective on oesophageal cancer: two diseases in one. Lancet Gastroenterol Hepatol. 2020;5:521–2. 32246940 10.1016/S2468-1253(20)30047-9

[7] Chen D, Su H, Li Y, Wu X, Li Y, Wei C, et al. miR-20b and miR-125a promote tumorigenesis in radioresistant esophageal carcinoma cells. Aging. 2021;13:9566–81. 33714953 10.18632/aging.202690 PMC8064182

[8] Wang J, Ma X, Si H, Ma Z, Ma Y, Wang J, et al. Role of long non-coding RNA H19 in therapy resistance of digestive system cancers. Mol Med. 2021;27:1. 33402118 10.1186/s10020-020-00255-2 PMC7786989

[9] Donlon NE, Davern M, O’Connell F, Sheppard A, Heeran A, Bhardwaj A, et al. Impact of radiotherapy on the immune landscape in oesophageal adenocarcinoma. World J Gastroenterol. 2022;28:2302–19. 35800186 10.3748/wjg.v28.i21.2302 PMC9185220

[10] Smit JK, Faber H, Niemantsverdriet M, Baanstra M, Bussink J, Hollema H, et al. Prediction of response to radiotherapy in the treatment of esophageal cancer using stem cell markers. Radiother Oncol. 2013;107:434–41. 23684587 10.1016/j.radonc.2013.03.027

[11] Penning TM, Jonnalagadda S, Trippier PC, Rižner TL. Aldo-keto reductases and cancer drug resistance. Pharmacol Rev. 2021;73:1150–71. 34312303 10.1124/pharmrev.120.000122 PMC8318518

[12] Jez JM, Penning TM. The aldo-keto reductase (AKR) superfamily: an update. Chem Biol Interact. 2001;130-132:499–525. 11306071 10.1016/s0009-2797(00)00295-7

[13] Jez JM, Flynn TG, Penning TM. A new nomenclature for the aldo-keto reductase superfamily. Biochem Pharm. 1997;54:639–47. 9310340 10.1016/s0006-2952(97)84253-0

[14] Suzuki-Yamamoto T, Nishizawa M, Fukui M, Okuda-Ashitaka E, Nakajima T, Ito S, et al. cDNA cloning, expression and characterization of human prostaglandin F synthase. FEBS Lett. 1999;462:335–40. 10622721 10.1016/s0014-5793(99)01551-3

[15] Zhou Q, Tian W, Jiang Z, Huang T, Ge C, Liu T, et al. A positive feedback loop of AKR1C3-mediated activation of NF-κB and STAT3 facilitates proliferation and metastasis in hepatocellular carcinoma. Cancer Res. 2021;81:1361–74. 33361392 10.1158/0008-5472.CAN-20-2480

[16] Wu C, Dai C, Li X, Sun M, Chu H, Xuan Q, et al. AKR1C3-dependent lipid droplet formation confers hepatocellular carcinoma cell adaptability to targeted therapy. Theranostics. 2022;12:7681–98. 36451864 10.7150/thno.74974 PMC9706585

[17] Liu Y, Chen Y, Jiang J, Chu X, Guo Q, Zhao L, et al. Development of highly potent and specific AKR1C3 inhibitors to restore the chemosensitivity of drug-resistant breast cancer. Eur J Med Chem. 2023;247:115013. 36566714 10.1016/j.ejmech.2022.115013

[18] Hertzog JR, Zhang Z, Bignan G, Connolly PJ, Heindl JE, Janetopoulos CJ, et al. AKR1C3 mediates pan-AR antagonist resistance in castration-resistant prostate cancer. Prostate. 2020;80:1223–32. 33258507 10.1002/pros.24049

[19] Xiong W, Zhao J, Yu H, Li X, Sun S, Li Y, et al. Elevated expression of AKR1C3 increases resistance of cancer cells to ionizing radiation via modulation of oxidative stress. PLoS ONE. 2014;9:e111911. 25419901 10.1371/journal.pone.0111911 PMC4242615

[20] Zhou C, Wang Z, Li J, Wu X, Fan N, Li D, et al. Aldo-keto reductase 1C3 mediates chemotherapy resistance in esophageal adenocarcinoma via ROS detoxification. Cancers. 2021;13:2403. 34065695 10.3390/cancers13102403 PMC8156851

[21] Li M, Zhang L, Yu J, Wang X, Cheng L, Ma Z, et al. AKR1C3 in carcinomas: from multifaceted roles to therapeutic strategies. Front Pharm. 2024;15:1378292. 10.3389/fphar.2024.1378292 PMC10957692 38523637

[22] Zhang C, Liu X, Jin S, Chen Y, Guo R. Ferroptosis in cancer therapy: a novel approach to reversing drug resistance. Mol Cancer. 2022;21:47. 35151318 10.1186/s12943-022-01530-y PMC8840702

[23] Chen X, Kang R, Kroemer G, Tang D. Broadening horizons: the role of ferroptosis in cancer. Nat Rev Clin Oncol. 2021;18:280–96. 33514910 10.1038/s41571-020-00462-0

[24] Xie Y, Hou W, Song X, Yu Y, Huang J, Sun X, et al. Ferroptosis: process and function. Cell Death Differ. 2016;23:369–79. 26794443 10.1038/cdd.2015.158 PMC5072448

[25] Bersuker K, Hendricks JM, Li Z, Magtanong L, Ford B, Tang PH, et al. The CoQ oxidoreductase FSP1 acts parallel to GPX4 to inhibit ferroptosis. Nature. 2019;575:688–92. 31634900 10.1038/s41586-019-1705-2 PMC6883167

[26] Yang WS, SriRamaratnam R, Welsch ME, Shimada K, Skouta R, Viswanathan VS, et al. Regulation of ferroptotic cancer cell death by GPX4. Cell. 2014;156:317–31. 24439385 10.1016/j.cell.2013.12.010 PMC4076414

[27] Mao C, Liu X, Zhang Y, Lei G, Yan Y, Lee H, et al. DHODH-mediated ferroptosis defence is a targetable vulnerability in cancer. Nature. 2021;593:586–90. 33981038 10.1038/s41586-021-03539-7 PMC8895686

[28] Doll S, Freitas FP, Shah R, Aldrovandi M, da Silva MC, Ingold I, et al. FSP1 is a glutathione-independent ferroptosis suppressor. Nature. 2019;575:693–8. 31634899 10.1038/s41586-019-1707-0

[29] Chen X, Li J, Kang R, Klionsky DJ, Tang D. Ferroptosis: machinery and regulation. Autophagy. 2021;17:2054–81. 32804006 10.1080/15548627.2020.1810918 PMC8496712

[30] Zhang W, Sun Y, Bai L, Zhi L, Yang Y, Zhao Q, et al. RBMS1 regulates lung cancer ferroptosis through translational control of SLC7A11. J Clin Investig. 2021;131:e152067. 34609966 10.1172/JCI152067 PMC8592553

[31] Chen Q, Zheng W, Guan J, Liu H, Dan Y, Zhu L, et al. SOCS2-enhanced ubiquitination of SLC7A11 promotes ferroptosis and radiosensitization in hepatocellular carcinoma. Cell Death Differ. 2023;30:137–51. 35995846 10.1038/s41418-022-01051-7 PMC9883449

[32] Lei G, Zhang Y, Koppula P, Liu X, Zhang J, Lin SH, et al. The role of ferroptosis in ionizing radiation-induced cell death and tumor suppression. Cell Res. 2020;30:146–62. 31949285 10.1038/s41422-019-0263-3 PMC7015061

[33] Jiang K, Yin X, Zhang Q, Yin J, Tang Q, Xu M, et al. STC2 activates PRMT5 to induce radioresistance through DNA damage repair and ferroptosis pathways in esophageal squamous cell carcinoma. Redox Biol. 2023;60:102626. 36764215 10.1016/j.redox.2023.102626 PMC9929488

[34] Yang M, Wu X, Hu J, Wang Y, Wang Y, Zhang L, et al. COMMD10 inhibits HIF1α/CP loop to enhance ferroptosis and radiosensitivity by disrupting Cu-Fe balance in hepatocellular carcinoma. J Hepatol. 2022;76:1138–50. 35101526 10.1016/j.jhep.2022.01.009

[35] Chen J, Zhang J, Tian W, Ge C, Su Y, Li J, et al. AKR1C3 suppresses ferroptosis in hepatocellular carcinoma through regulation of YAP/SLC7A11 signaling pathway. Mol Carcinog. 2023;62:833–44. 36920042 10.1002/mc.23527

[36] Elkind MM, Sutton H. Radiation response of mammalian cells grown in culture I. Repair of X-ray damage in surviving Chinese hamster cells. 1960 Radiat Res. 2012;178:Av8–26. 22870981 10.1667/rrav02.1

[37] Detre S, Saclani Jotti G, Dowsett M. A “quickscore” method for immunohistochemical semiquantitation: validation for oestrogen receptor in breast carcinomas. J Clin Pathol. 1995;48:876–8. 7490328 10.1136/jcp.48.9.876 PMC502883

[38] Fan N, Raatz L, Chon SH, Quaas A, Bruns C, Zhao Y. Subculture and cryopreservation of esophageal adenocarcinoma organoids: pros and cons for single cell digestion. J Vis Exp. 2022;6:185 10.3791/63281 35876535

[39] Lynam-Lennon N, Reynolds JV, Pidgeon GP, Lysaght J, Marignol L, Maher SG. Alterations in DNA repair efficiency are involved in the radioresistance of esophageal adenocarcinoma. Radiat Res. 2010;174:703–11. 21128793 10.1667/RR2295.1

[40] Bonner WM, Redon CE, Dickey JS, Nakamura AJ, Sedelnikova OA, Solier S, et al. GammaH2AX and cancer. Nat Rev Cancer. 2008;8:957–67. 19005492 10.1038/nrc2523 PMC3094856

[41] Mah LJ, El-Osta A, Karagiannis TC. gammaH2AX: a sensitive molecular marker of DNA damage and repair. Leukemia. 2010;24:679–86. 20130602 10.1038/leu.2010.6

[42] Olive PL, Banáth JP. The comet assay: a method to measure DNA damage in individual cells. Nat Protoc. 2006;1:23–29. 17406208 10.1038/nprot.2006.5

[43] Dixon SJ, Lemberg KM, Lamprecht MR, Skouta R, Zaitsev EM, Gleason CE, et al. Ferroptosis: an iron-dependent form of nonapoptotic cell death. Cell. 2012;149:1060–72. 22632970 10.1016/j.cell.2012.03.042 PMC3367386

[44] Byrns MC, Jin Y, Penning TM. Inhibitors of type 5 17β-hydroxysteroid dehydrogenase (AKR1C3): overview and structural insights. J Steroid Biochem Mol Biol. 2011;125:95–104. 21087665 10.1016/j.jsbmb.2010.11.004 PMC3047600

[45] Zhu S, Zhang Q, Sun X, Zeh HJ 3rd, Lotze MT, Kang R, et al. HSPA5 regulates ferroptotic cell death in cancer cells. Cancer Res. 2017;77:2064–77. 28130223 10.1158/0008-5472.CAN-16-1979 PMC5392369

[46] Mun SH, Lee CS, Kim HJ, Kim J, Lee H, Yang J, et al. Marchf6 E3 ubiquitin ligase critically regulates endoplasmic reticulum stress, ferroptosis, and metabolic homeostasis in POMC neurons. Cell Rep. 2023;42:112746. 37421621 10.1016/j.celrep.2023.112746

[47] Chen Y, Mi Y, Zhang X, Ma Q, Song Y, Zhang L, et al. Dihydroartemisinin-induced unfolded protein response feedback attenuates ferroptosis via PERK/ATF4/HSPA5 pathway in glioma cells. J Exp Clin Cancer Res. 2019;38:402. 31519193 10.1186/s13046-019-1413-7 PMC6743121

[48] Tuncer C, Hacioglu C. Borax induces ferroptosis of glioblastoma by targeting HSPA5/NRF2/GPx4/GSH pathways. J Cell Mol Med. 2024;28:e18206. 38494858 10.1111/jcmm.18206 PMC10945083

[49] Du T, Li H, Fan Y, Yuan L, Guo X, Zhu Q, et al. The deubiquitylase OTUD3 stabilizes GRP78 and promotes lung tumorigenesis. Nat Commun. 2019;10:2914. 31266968 10.1038/s41467-019-10824-7 PMC6606649

[50] Kim SY, Kim HJ, Kim HJ, Kim DH, Han JH, Byeon HK, et al. HSPA5 negatively regulates lysosomal activity through ubiquitination of MUL1 in head and neck cancer. Autophagy. 2018;14:385–403. 29260979 10.1080/15548627.2017.1414126 PMC5915028

[51] Zhang T, Li J, Yang M, Ma X, Wang Z, Ma X, et al. CDK7/GRP78 signaling axis contributes to tumor growth and metastasis in osteosarcoma. Oncogene. 2022;41:4524–36. 36042349 10.1038/s41388-022-02446-z

[52] Medvar B, Raghuram V, Pisitkun T, Sarkar A, Knepper MA. Comprehensive database of human E3 ubiquitin ligases: application to aquaporin-2 regulation. Physiol Genom. 2016;48:502–12. 10.1152/physiolgenomics.00031.2016 PMC4967219 27199454

[53] Wang J, Lee J, Liem D, Ping P. HSPA5 Gene encoding Hsp70 chaperone BiP in the endoplasmic reticulum. Gene. 2017;618:14–23. 28286085 10.1016/j.gene.2017.03.005 PMC5632570

[54] Rogers JE, Sewastjanow-Silva M, Waters RE, Ajani JA. Esophageal cancer: emerging therapeutics. Expert Opin Ther Targets. 2022;26:107–17. 35119973 10.1080/14728222.2022.2036718

[55] Carraro A, Trevellin E, Fassan M, Kotsafti A, Lunardi F, Porzionato A, et al. Esophageal adenocarcinoma microenvironment: peritumoral adipose tissue effects associated with chemoresistance. Cancer Sci. 2017;108:2393–404. 28985034 10.1111/cas.13415 PMC5715298

[56] Buckley AM, Dunne MR, Lynam-Lennon N, Kennedy SA, Cannon A, Reynolds AL. et al. Pyrazinib (P3), [(E)-2-(2-Pyrazin-2-yl-vinyl)-phenol], a small molecule pyrazine compound enhances radiosensitivity in oesophageal adenocarcinoma. Cancer Lett. 2019;447:115–29. 30664962 10.1016/j.canlet.2019.01.009

[57] Li X, Hong X, Gao X, Gu X, Xiong W, Zhao J, et al. Methyl jasmonate enhances the radiation sensitivity of esophageal carcinoma cells by inhibiting the 11-ketoprostaglandin reductase activity of AKR1C3. Cancer Manag Res. 2018;10:3149–58. 30214307 10.2147/CMAR.S166942 PMC6124458

[58] Xiong W, Huang X, Yao S, Wang L, Wang J, Wang X. Expression of AKR1C3, β-Catenin and LEF1 in esophageal squamous cell carcinoma and the relationship with radiation resistance. Iran J Public Health. 2021;50:1488–90. 34568190 10.18502/ijph.v50i7.6641 PMC8426780

[59] Srinivas US, Tan BWQ, Vellayappan BA, Jeyasekharan AD. ROS and the DNA damage response in cancer. Redox Biol. 2019;25:101084. 30612957 10.1016/j.redox.2018.101084 PMC6859528

[60] Dixon SJ, Patel DN, Welsch M, Skouta R, Lee ED, Hayano M, et al. Pharmacological inhibition of cystine-glutamate exchange induces endoplasmic reticulum stress and ferroptosis. Elife. 2014;3:e02523. 24844246 10.7554/eLife.02523 PMC4054777

[61] TeSlaa T, Ralser M, Fan J, Rabinowitz JD. The pentose phosphate pathway in health and disease. Nat Metab. 2023;5:1275–89. 37612403 10.1038/s42255-023-00863-2 PMC11251397

[62] Wang L, Li D, Su X, Zhao Y, Huang A, Li H, et al. AGO4 suppresses tumor growth by modulating autophagy and apoptosis via enhancing TRIM21-mediated ubiquitination of GRP78 in a p53-independent manner. Oncogene. 2023;42:62–77. 36371565 10.1038/s41388-022-02526-0

[63] Peraldo-Neia C, Ostano P, Mello-Grand M, Guana F, Gregnanin I, Boschi D, et al. AKR1C3 is a biomarker and druggable target for oropharyngeal tumors. Cell Oncol. 2021;44:357–72. 10.1007/s13402-020-00571-z PMC12980764 33211282

[64] Reddi D, Seaton BW, Woolston D, Aicher L, Monroe LD, Mao ZJ, et al. AKR1C3 expression in T acute lymphoblastic leukemia/lymphoma for clinical use as a biomarker. Sci Rep. 2022;12:5809. 35388063 10.1038/s41598-022-09697-6 PMC8986791

[65] Moradi Manesh D, El-Hoss J, Evans K, Richmond J, Toscan CE, Bracken LS, et al. AKR1C3 is a biomarker of sensitivity to PR-104 in preclinical models of T-cell acute lymphoblastic leukemia. Blood. 2015;126:1193–202. 26116659 10.1182/blood-2014-12-618900 PMC4559932

[66] Tian Y, Zhao L, Zhang H, Liu X, Zhao L, Zhao X, et al. AKR1C3 overexpression may serve as a promising biomarker for prostate cancer progression. Diagn Pathol. 2014;9:42. 24571686 10.1186/1746-1596-9-42 PMC3939640

[67] Meng F, Li WF, Jung D, Wang CC, Qi T, Shia CS, et al. A novel selective AKR1C3-activated prodrug AST-3424/OBI-3424 exhibits broad anti-tumor activity. Am J Cancer Res. 2021;11:3645–59. 34354865 PMC8332853

[68] Tsimberidou AM, Verschraegen CF, Wesolowski R, Shia CS, Hsu P, Pearce TE. Phase 1 dose-escalation study evaluating the safety, pharmacokinetics, and clinical activity of OBI-3424 in patients with advanced or metastatic solid tumors. Br J Cancer. 2023;129:266–74. 37173365 10.1038/s41416-023-02280-4 PMC10180615

[69] Meng T, Jung D, Cai XH, Lu ZQ, Yu JB, Qi TY, et al. Characterization of AST-001 non-clinical pharmacokinetics: a novel selective AKR1C3-activated prodrug in mice, rats, and cynomolgus monkeys. Biopharm Drug Dispos. 2024;45:83–92. 38492211 10.1002/bdd.2385

