# Supplementary material for: AKR1C3 enhances radioresistance in esophageal adenocarcinoma via inhibiting ferroptosis through suppressing TRIM21-mediated ubiquitination of HSPA5
Source: Cell Death Dis. 2025 Jul 2;16(1):483. doi: 10.1038/s41419-025-07773-z (PMC12222831; doi:10.1038/s41419-025-07773-z)
Supplement: Supplementary file 2 — Supplementary data [file 41419_2025_7773_MOESM2_ESM.doc]

**Supplementary Data**

**The Supplementary Data consist of:**

Supplementary Fig. S1-4

Supplementary Table. S1-2


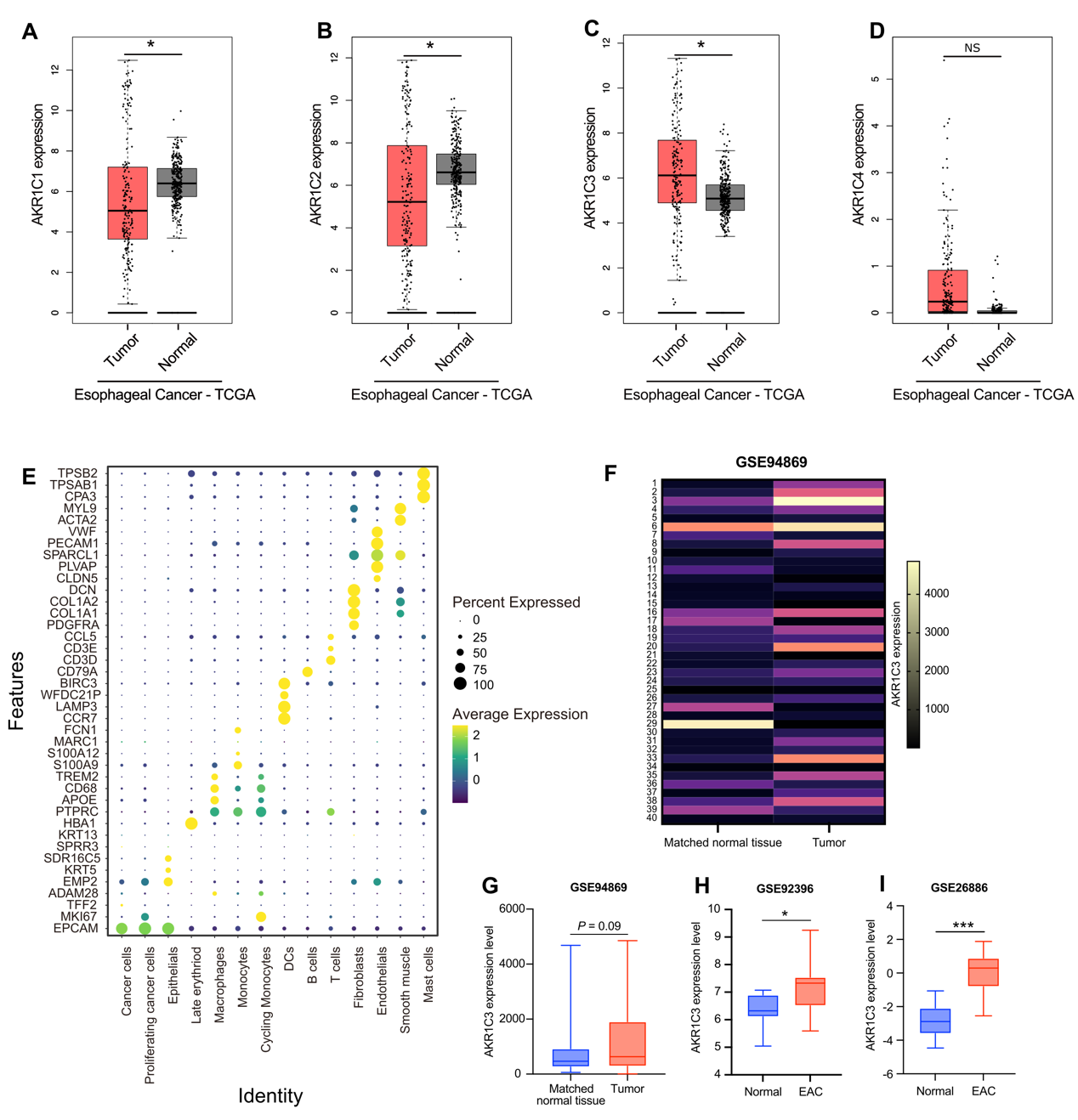


**Fig. S1.** **AKR1C3 is associated with therapeutic response and prognosis of EAC patients.** **A-D** AKR1C family genes expression levels in TCGA esophageal cancer cohort. **E** Dotplot exhibited the top cluster marker genes, with dot size indicating the percentage of the cluster expression. **F-I** Data from the GEO datasets showed AKR1C3 expression level is higher in EAC tissues compared to the adjacent normal tissues. Statistical comparisons were made using a paired two-tailed Student’s t-test; **P* < 0.05, ***P* < 0.01, ****P* < 0.001.


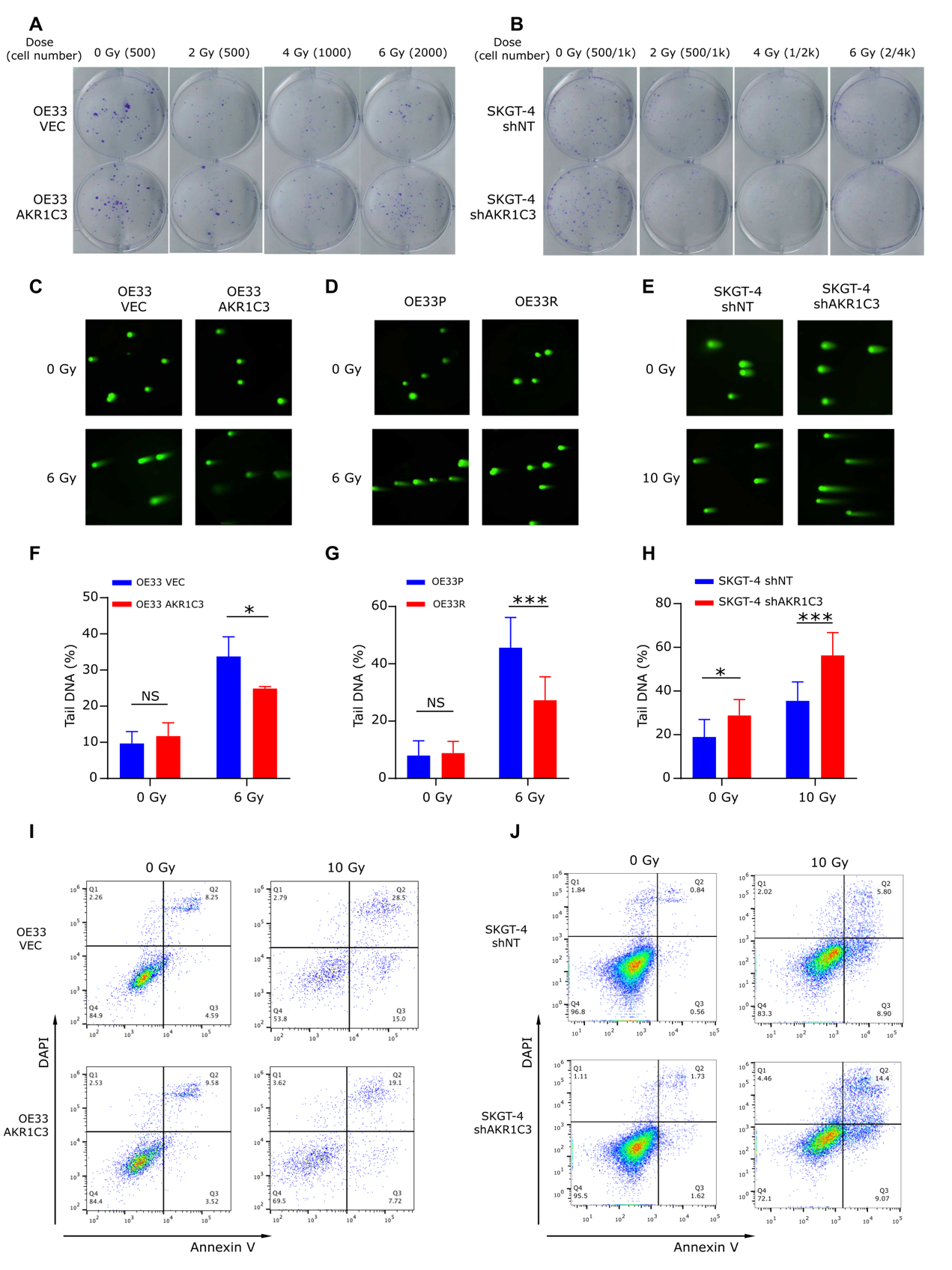


**Fig. S2.** **AKR1C3 could enhance the radioresistance in EAC cells. A-B** Survival fraction after 0 - 6 Gy irradiation in OE33 VEC / OE33 AKR1C3 and SKGT-4 shNC / SKGT-4 shAKR1C3. To ensure an adequate number of colonies for SKGT-4 shAKR1C3 after exposure to 6 Gy, the cell seeding number in SKGT-4 shAKR1C3 was doubled compared to SKGT-4 shNC. **C-H** Cells were treated with irradiation (6 Gy for OE33 and 10 Gy for SKGT-4). 4h after irradiation, cells were harvested and then electrophoresed. Nuclei were stained with Nancy-520. Bar charts presented the percentage of Tail DNA in each group. Means ± SD, N ≥ 5. **I-J** Cells were treated with 0 or 10 Gy irradiation. Flow cytometry was performed 72h after treatment. Dot plots showed the dead cells before or after irradiation in OE33 VEC / OE33 AKR1C3 and SKGT-4 shNC / SKGT-4 shAKR1C3 by Annexin V and DAPI staining. Statistical comparisons were made using a paired two-tailed Student’s t-test; **P* < 0.05, ***P* < 0.01, ****P* < 0.001.


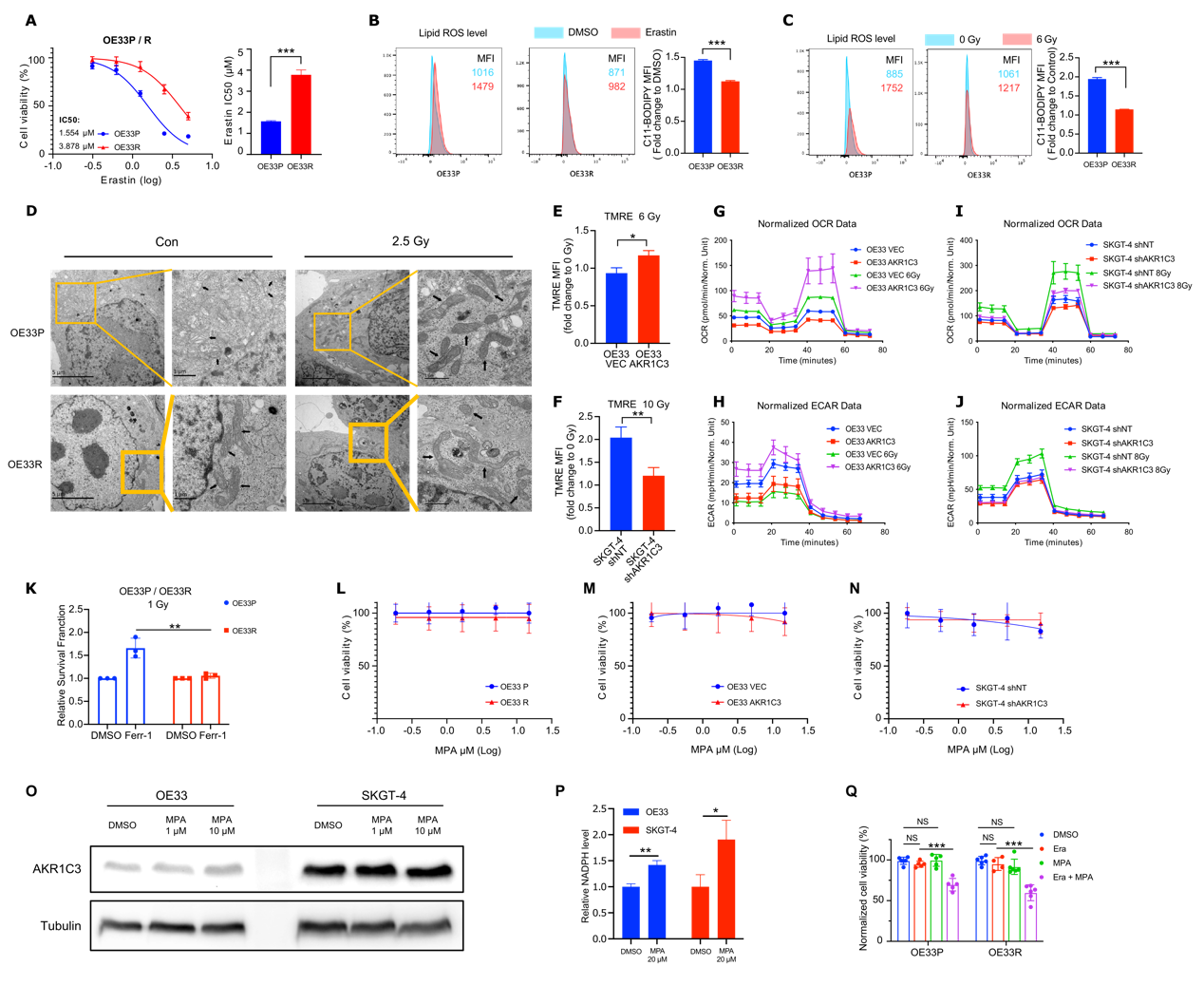


**Fig. S3.** **AKR1C3 regulates redox homeostasis and inhibits ferroptosis in EAC cells.** **A** OE33 P / R cells were treated with 0.3125 - 5 µM erastin for 72h. The relative cell viability was measured by the MTT assay. **B** Lipid peroxidation level was detected by C11-Bodipy staining on flow cytometry. The concentration of C11-Bodipy for staining was 1 µM. OE33 cells were treated with 1 µM erastin for 48h. Bar graphs showing erastin-induced relative fold change of lipid peroxidation levels. **C** OE33P / R cells were treated with 6 Gy. After 48h, lipid peroxidation level was detected by C11-Bodipy staining on flow cytometry. The concentration of C11-Bodipy for staining was 1 µM. Bar graphs showing irradiation-induced relative fold change of lipid peroxidation levels. **D** TEM images of OE33P / OE33R before and after radiotherapy (2.5 Gy, fixation after 24h). Black arrow: mitochondria. A minimum of five cells in each group were examined. **E, F** TMRE was stained for detecting mitochondrial activity. Bar charts showed the fold change of the TMRE MFI before and after radiotherapy. **G-J** The EAC cells in treatment groups were treated with 6-8 Gy irradiation. Seahorse experiments were performed 24h after treatment. In the cell mito stress test, Oligomycin (at 20min), FCCP (at 40min), and Rotenone & Antimycin A (at 60min) were added to the reaction separately. In the glycolytic rate assay, Rotenone & Antimycin A (at 20min) and 2-deoxy-D-glucose (at 40min) were added to the reaction separately. **K** 500 cells were seeded in the 6-well plated, 1 Gy irradiation on the second day, the concentration of Ferr-1 was 0.3 µM. Cells were fixed and counted after 7-12 days. Data was normalized with the DMSO groups. **L-N** OE33P / R, OE33 VEC / AKR1C3 and SKGT-4 shNT / shAKR1C3 cells were treated with MPA (up to 15 µM) for 72h. The relative cell viability was measured by the MTT assay. Data was normalized with the DMSO groups. **O** OE33 and SKGT-4 cells were treated with 1 and 10 µM MPA for 72h. DMSO set as a control. Protein level was measured by Western blot. **P** OE33 and SKGT-4 cells were seeded overnight. Medium were changed with glucose/glutamine-free medium to block NADPH synthesis, and 10 µM MPA was added for 2 hours. The luminescence of NADPH was measured by the luminometer 30min after incubation with NADPH-Glo™ Detection Reagent. **Q** OE33 P/ R cells were treated with 1 µM erastin, 10 µM MPA and combined use of erastin / MPA for 48h. The relative cell viability was measured by the MTT assay. Data was normalized with the DMSO groups. Mean ± SD, N=3. * *P* < 0.05, ** *P* < 0.01, *** *P* < 0.001.


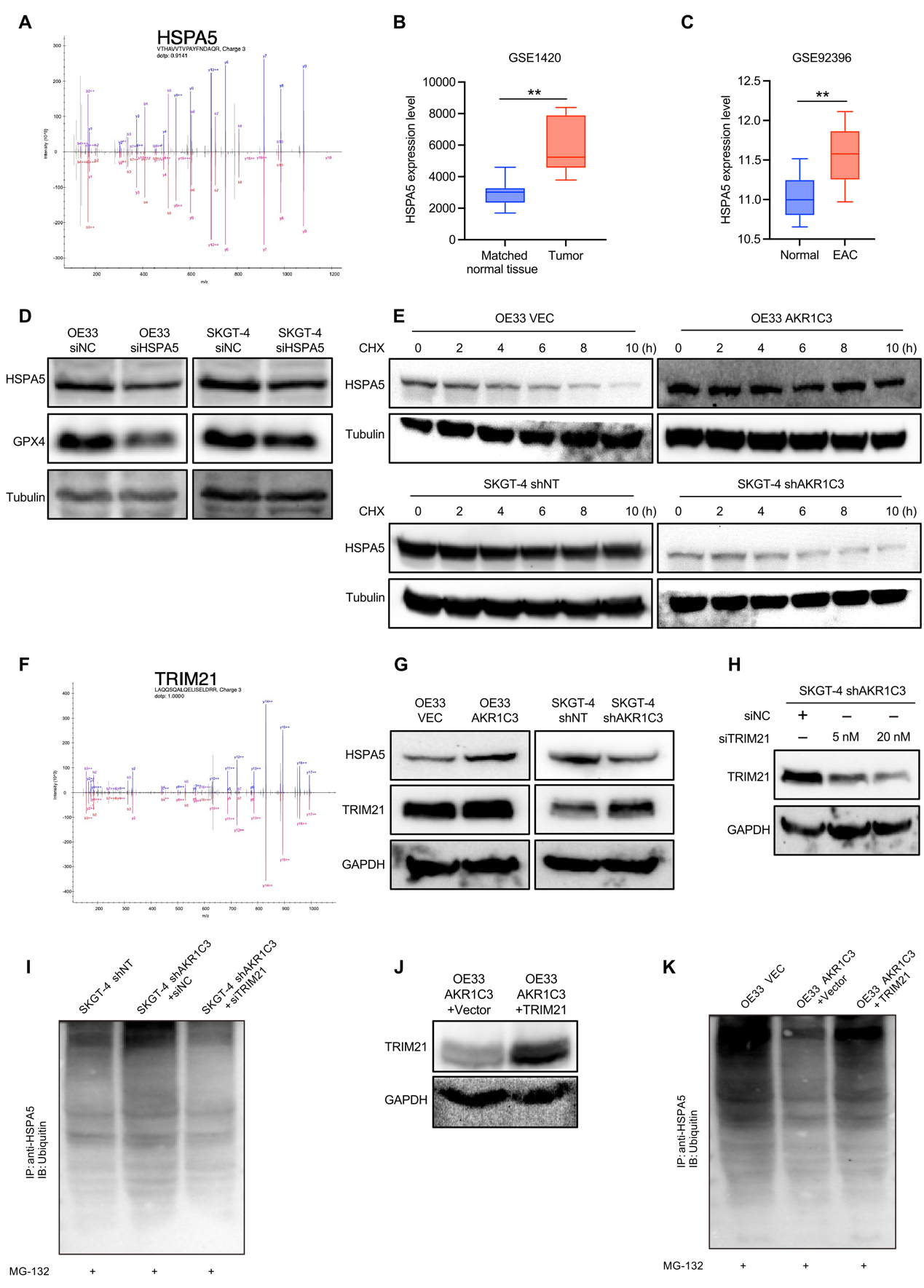


**Fig. S4. AKR1C3 stabilizes HSPA5/GPX4 axis to inhibit ferroptosis. A** The peptide spectrum of HSPA5 was determined by mass spectrometry in the anti-AKR1C3 immunoprecipitates of SKGT-4 cells. **B-C** Data from the GEO datasets showed HSPA5 expression levels are higher in EAC tissues compared to the adjacent normal tissues. Mean ± SD, * P < 0.05, ** P < 0.01. **D** Western blot results exhibited the protein expression of HSPA5 and GPX4 in different EAC cell lines. **E** Western blot results exhibited the protein expression of HSPA5 in different EAC cell lines with different co-culture time of cycloheximide. **F** the peptide spectrum of TRIM21 was determined by mass spectrometry in the anti-HSPA5 immunoprecipitates of OE33 AKR1C3. **G** Western blot results exhibited the protein expression of TRIM21 and GPX4 in different EAC cell lines. **H** Western blot results exhibited the protein expression of TRIM21 in SKGT-4 shAKR1C3 with different concentrations of siTRIM21. **I** Western blot results exhibited ubiquitin level of HSPA5 in TRIM21-knockdown cell line. **J** Western blot results exhibited the protein expression of TRIM21 in OE33 AKR1C3 with pLVX puro TRIM21-GFP transfection. Empty vector plasmid was applied as a control. **K** Western blot results exhibited ubiquitin level of HSPA5 in TRIM21-overexpressed cell line.

**Supplementary table**

| **Supplementary Table S1.** Details of the antibodies | | | |
| --- | --- | --- | --- |
| **Antibody** | **Company** | **Catalog** | **Application** |
| α-tubulin | Cell signaling technology | 3873 | WB |
| AKR1C3 | R&D Systems | MAB7678 | WB |
| AKR1C3 | Proteintech | 11194-1-AP | IP |
| GADPH | Cell signaling technology | 97166 | WB |
| GPX4 | Abcam | ab125066 | WB |
| HSPA5 | Cell signaling technology | 3177 | WB, IP, IF |
| HSPA5 | Proteintech | 66574-1-Ig | WB, IP, IF |
| H2AX | ThermoFisher scientific | MA1-2022 | IF |
| anti-mouse 2nd antibody | Invitrogen | 31430 | WB |
| Anti-Rabbit IgG (Light-Chain Specific) | Cell signaling technology | 93702 | WB after IP |
| anti-rabbit 2nd antibody | Invitrogen | 31460 | WB |
| Ubiquitin (P4D1) | BioLegend | 646302 | WB |
| TRIM21 | Cell signaling technology | 92043 | WB, IP |
| Normal Rabbit IgG | Cell signaling technology | 2729S | IP |

**Supplementary Table 2.**

mRNA expression levels of AKR1C family in RNA-seq data.

|  | baseMean | log2FoldChange | lfcSE | stat | pvalue | padj |
| --- | --- | --- | --- | --- | --- | --- |
| AKR1C1 | 490.868307 | 3.653771106 | 0.27578161 | 13.2487842 | 4.59E-40 | 1.04E-37 |
| AKR1C2 | 527.908929 | 3.748835149 | 0.24267446 | 15.4480001 | 7.78E-54 | 3.18E-51 |
| AKR1C3 | 820.820998 | 3.375727451 | 0.19640933 | 17.1872053 | 3.31E-66 | 1.98E-63 |
| AKR1C4 | 29.1756199 | 3.249182526 | 0.75567940 | 4.29968386 | 1.71E-05 | 0.0001454 |
