# Supplementary material for: AKR1C3 enhances radioresistance in esophageal adenocarcinoma via inhibiting ferroptosis through suppressing TRIM21-mediated ubiquitination of HSPA5
Source: Cell Death Dis. 2025 Jul 2;16(1):483. doi: 10.1038/s41419-025-07773-z (PMC12222831; doi:10.1038/s41419-025-07773-z)

Fig. 1H

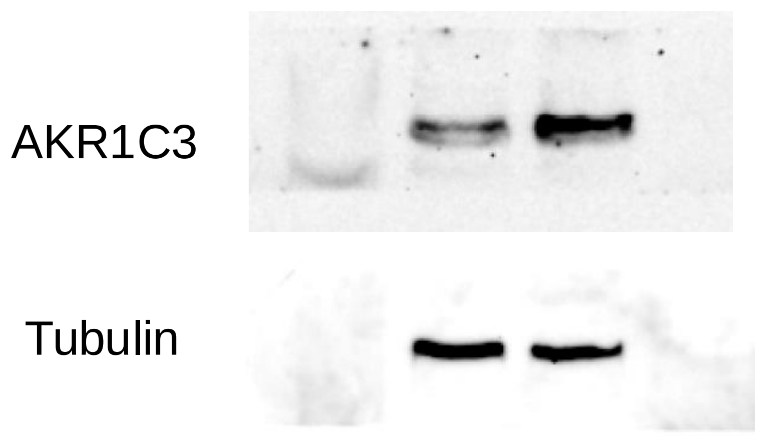

Fig. 3A

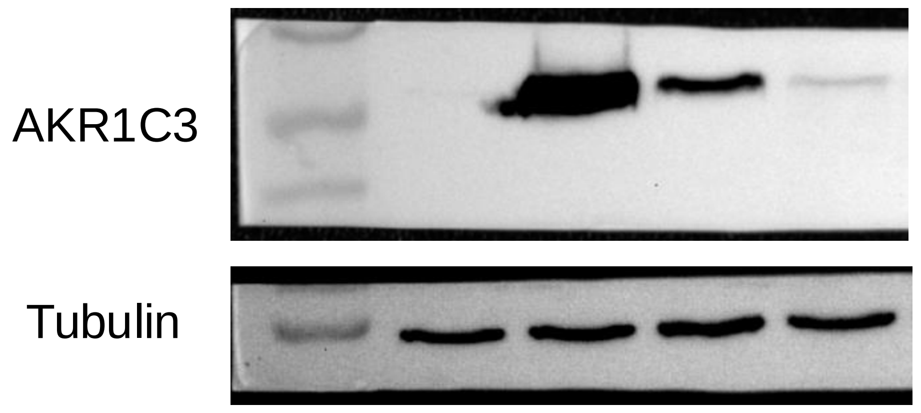

Fig. 3I

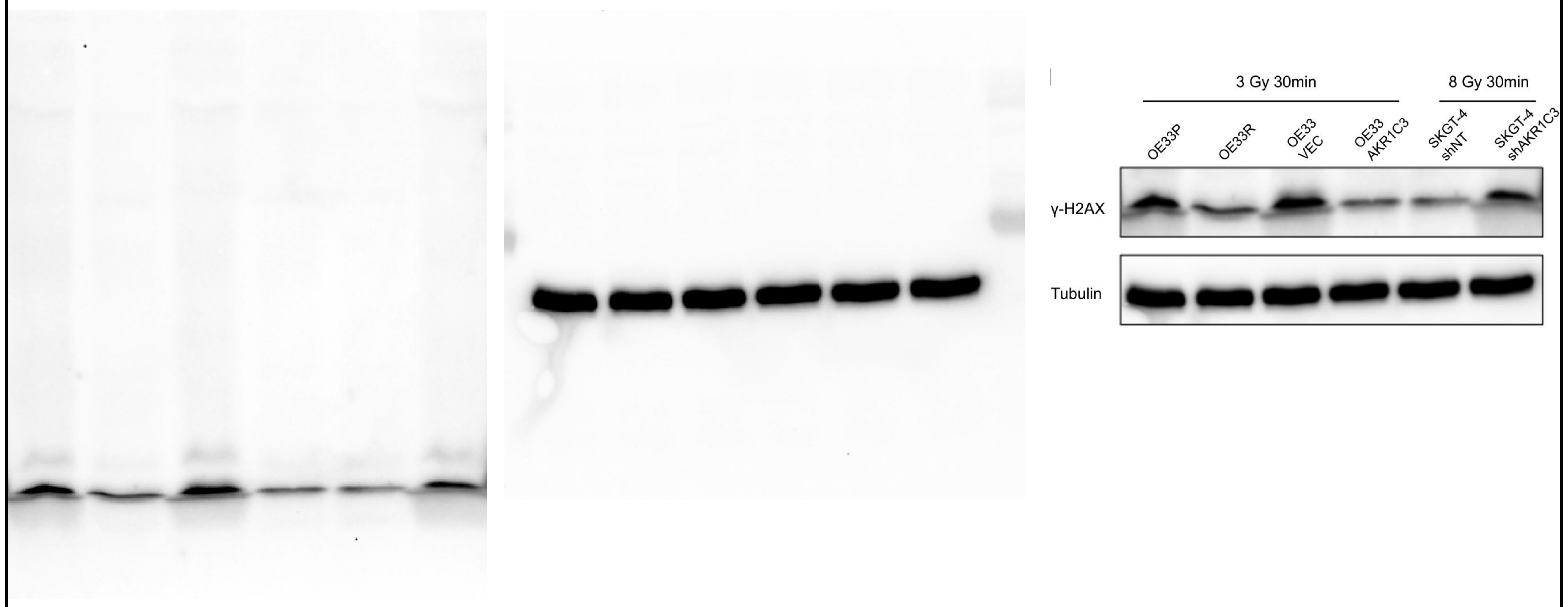

Fig. 6B

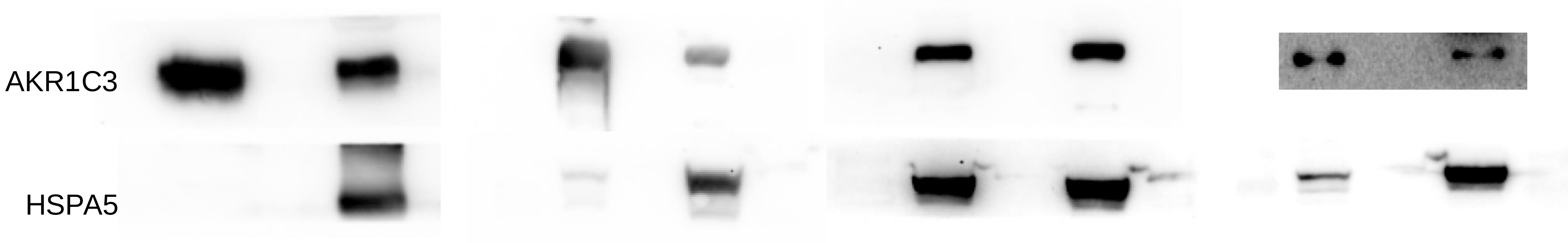

Fig. 6D

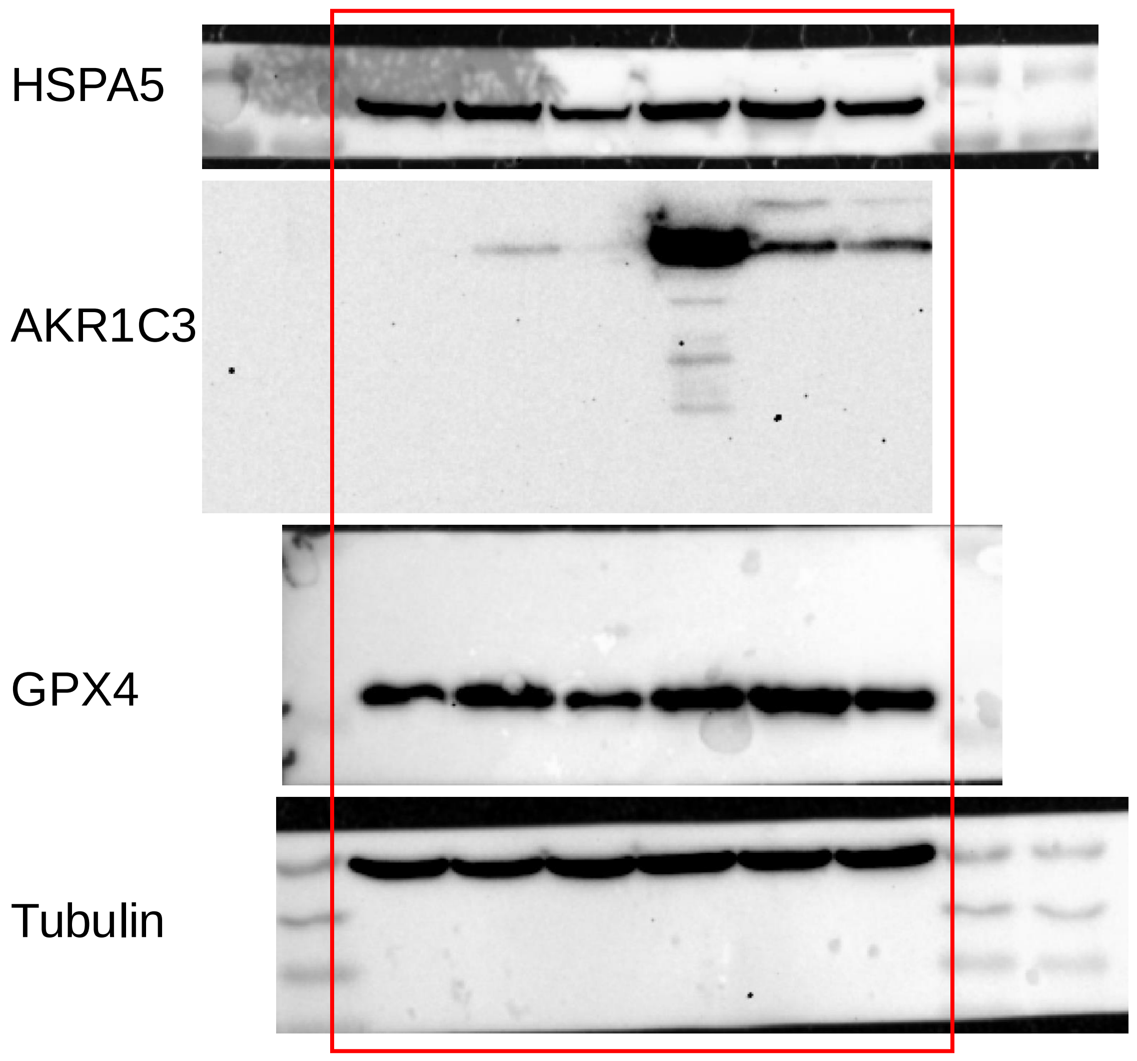

Fig. 6F

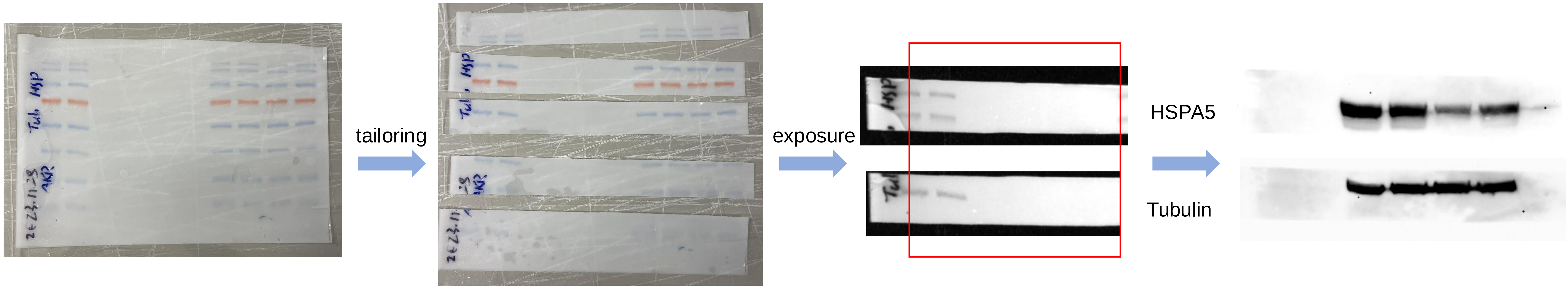

Fig. 6H

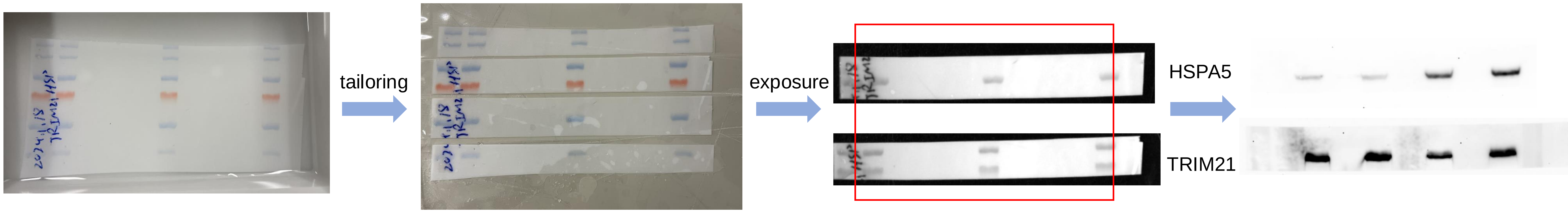

Fig. 6I

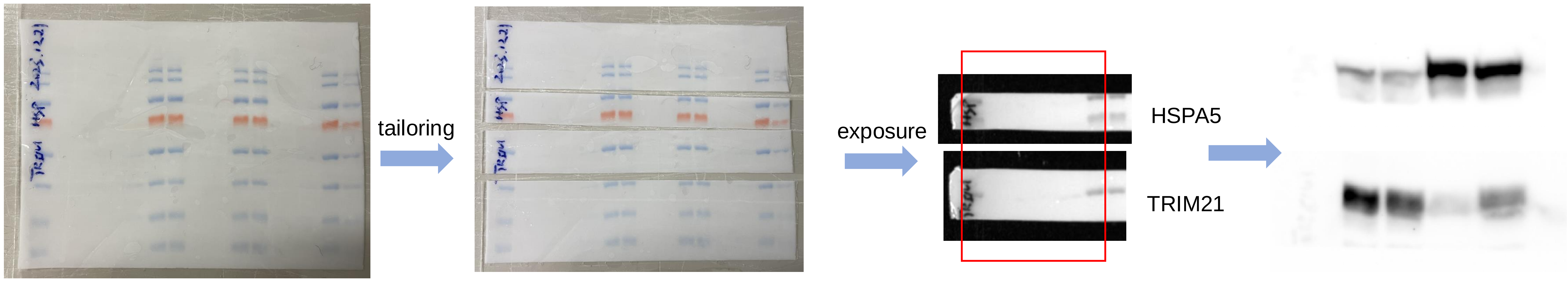

Fig. 6J

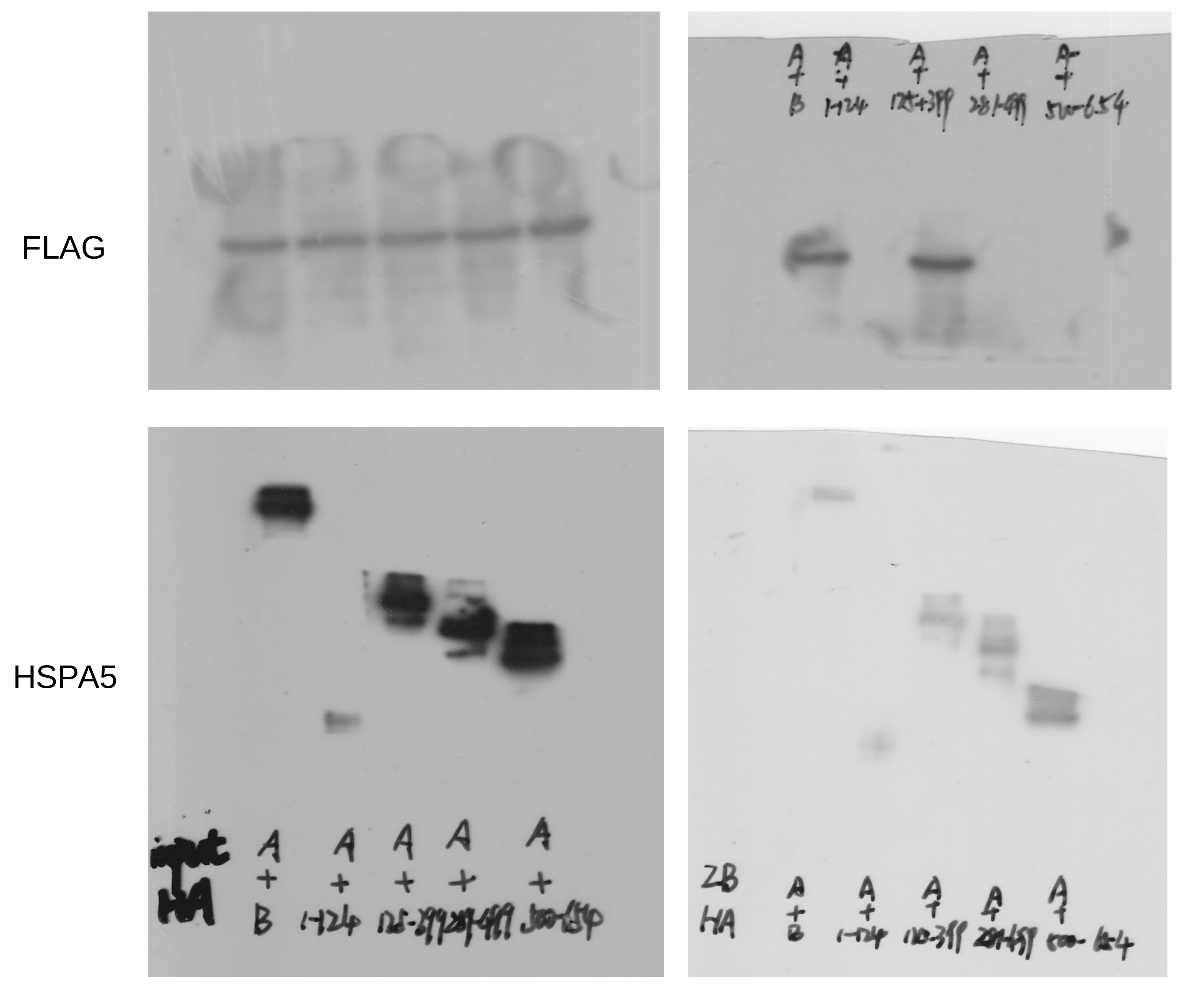

Fig. S3O

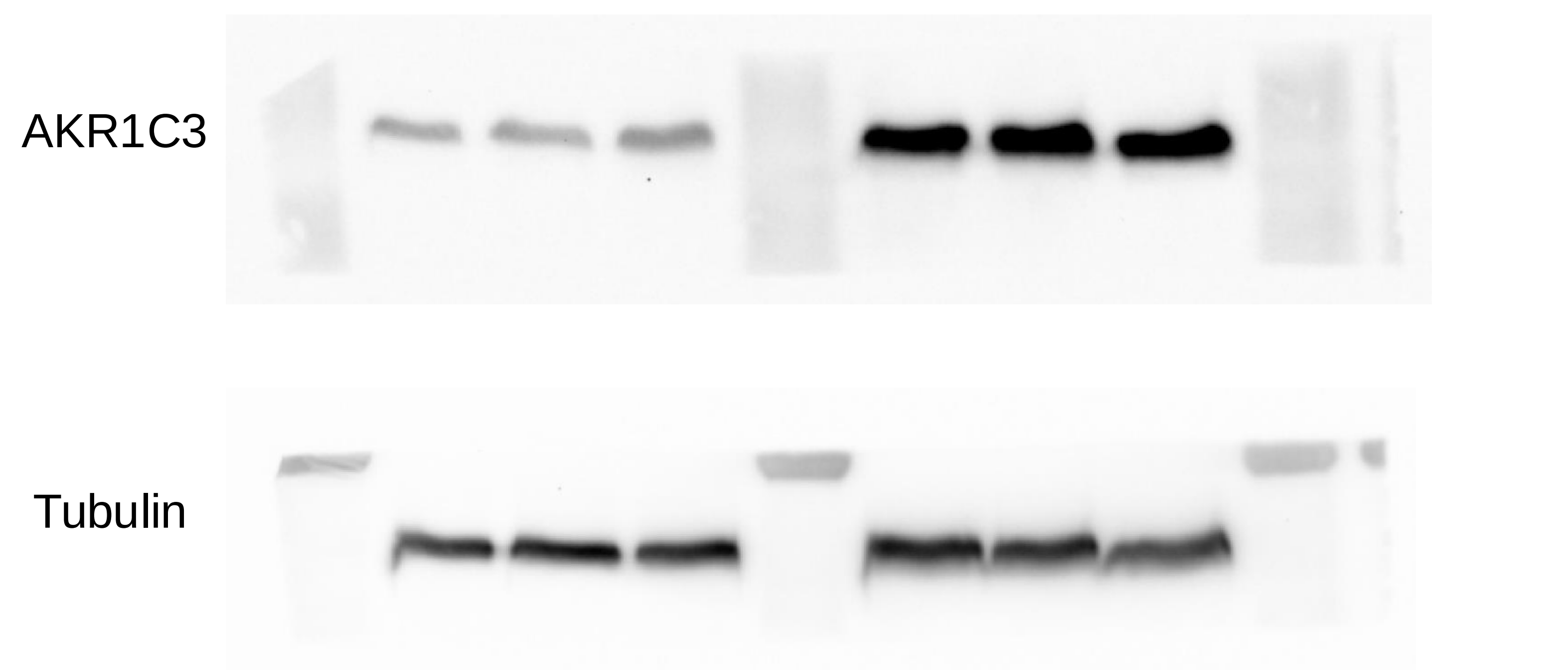

Fig. S4D

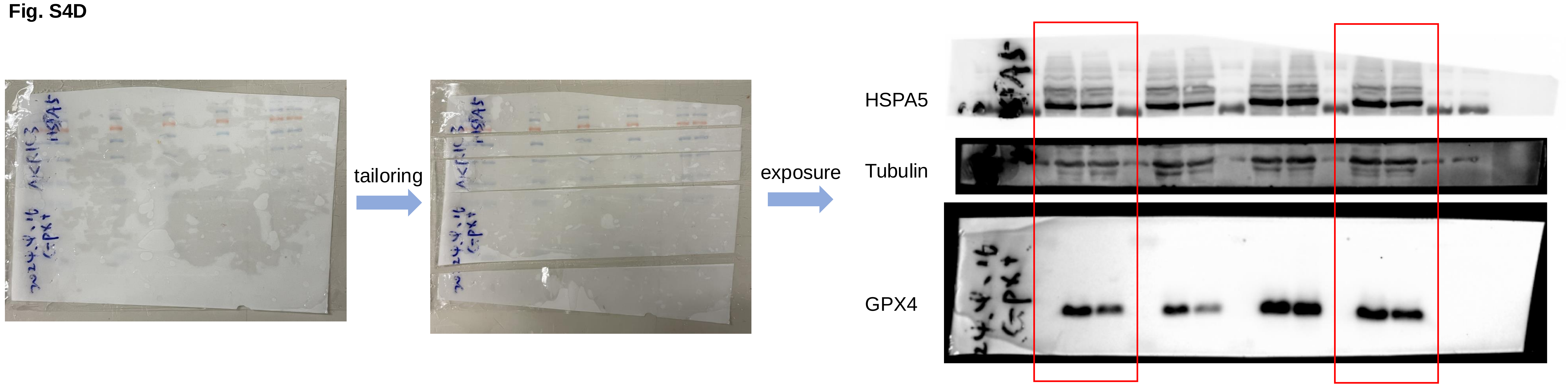

Fig.S4E

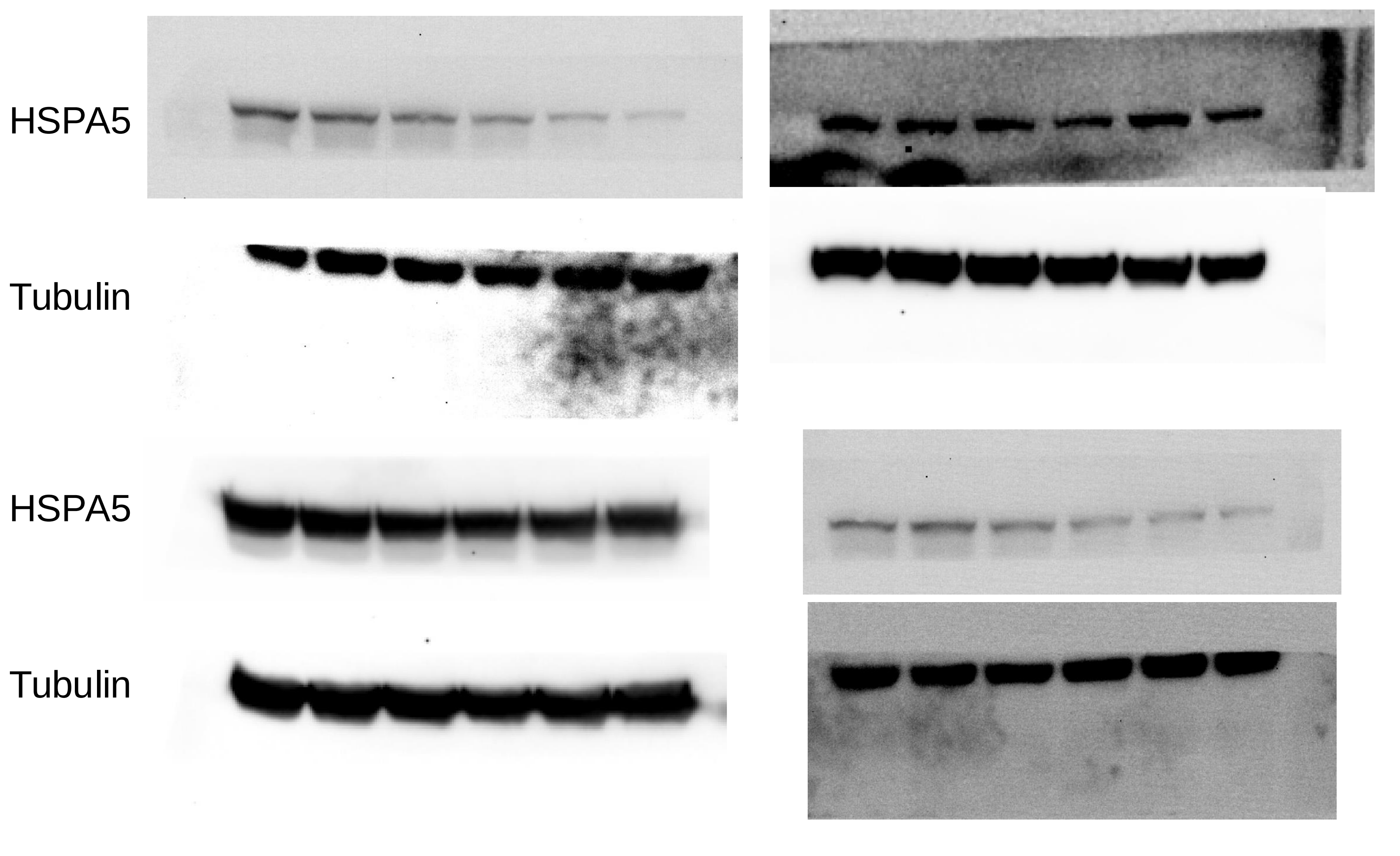

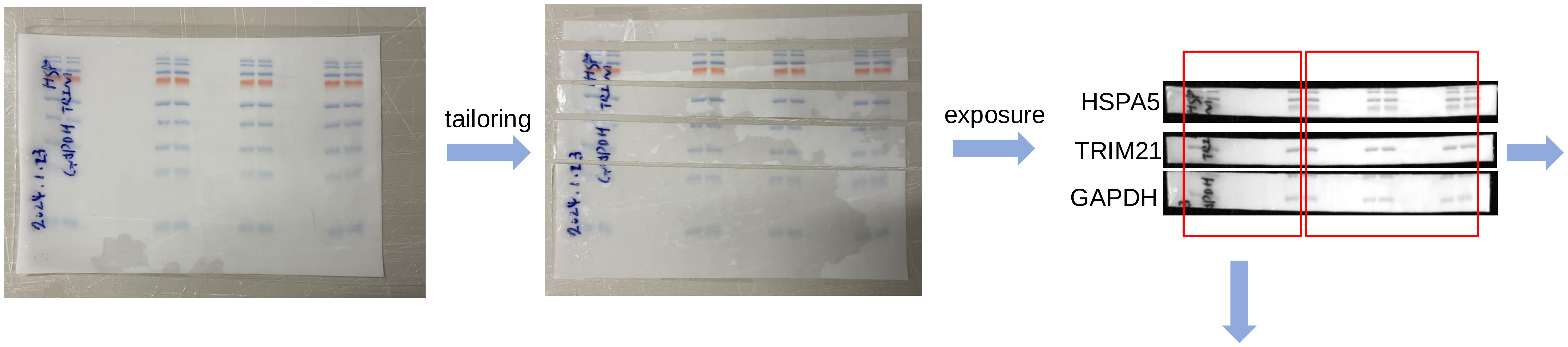

Fig. S4G

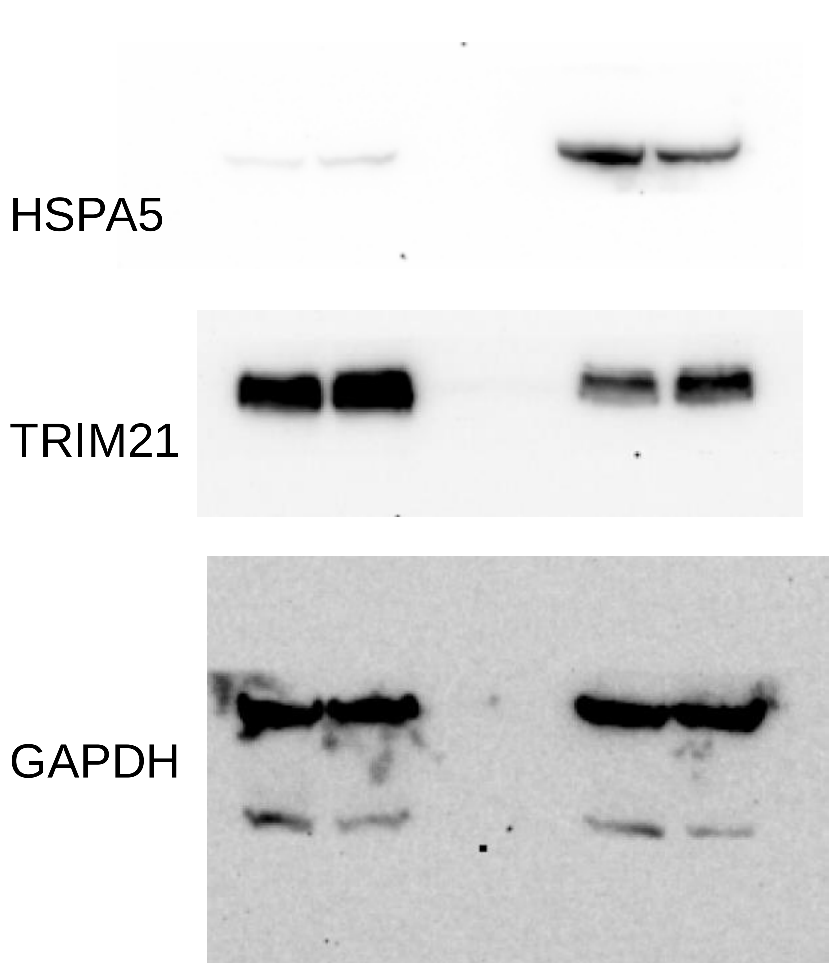

Fig. S4H

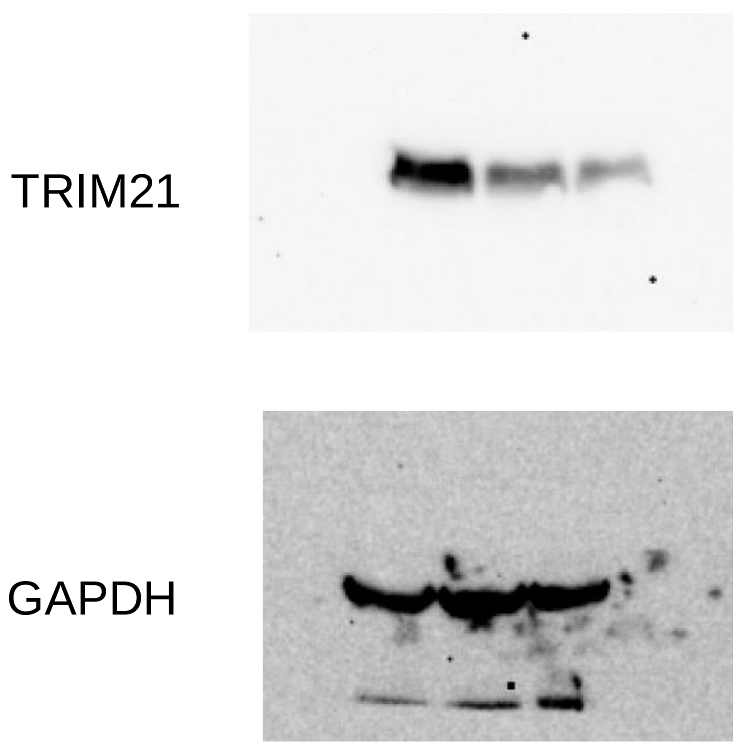

Fig. S4J

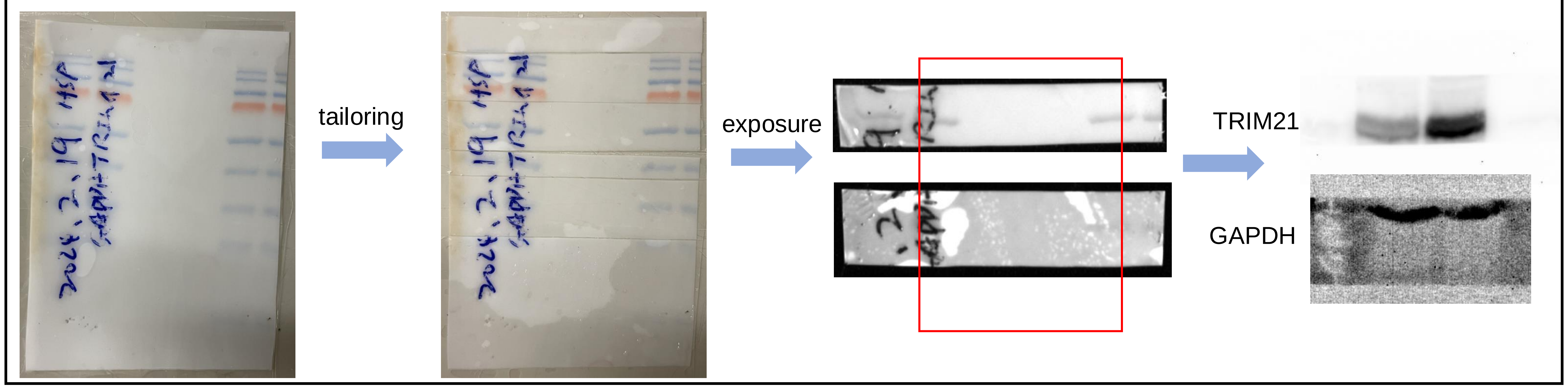

Supplement: Supplementary file 3 — Original blot [file 41419_2025_7773_MOESM3_ESM.pdf]
